# NLRP3 Sensing of Diverse Inflammatory Stimuli Requires Distinct Structural Features

**DOI:** 10.3389/fimmu.2020.01828

**Published:** 2020-08-26

**Authors:** Tabassum Rahman, Abhinit Nagar, Ellen B. Duffy, Kendi Okuda, Neal Silverman, Jonathan A. Harton

**Affiliations:** ^1^Department of Immunology and Microbial Disease, Albany Medical College, Albany, NY, United States; ^2^Division of Infectious Diseases and Immunology, Department of Medicine, University of Massachusetts Medical School, Worcester, MA, United States

**Keywords:** NLRP3, inflammasome, leucine-rich repeats (LRRs), Pyrin domain (PYD), cysteine, ROS, IL-1β, caspase-1

## Abstract

The NLRP3 inflammasome is central to host defense and implicated in various inflammatory diseases and conditions. While the favored paradigm of NLRP3 inflammasome activation stipulates a unifying signal intermediate that de-represses NLRP3, this view has not been tested. Further, structures within NLRP3 required for inflammasome activation are poorly defined. Here we demonstrate that while the NLRP3 LRRs are not auto-repressive and are not required for inflammasome activation by all agonists, distinct sequences within the NLRP3 LRRs positively and negatively modulate inflammasome activation by specific ligands. In addition, elements within the HD1/HD2 “hinge” of NLRP3 and the nucleotide-binding domain have contrasting functions depending upon the specific agonists. Further, while NLRP3 1–432 is minimally sufficient for inflammasome activation by all agonists tested, the pyrin, and linker domains (1–134) function cooperatively and are sufficient for inflammasome activation by certain agonists. Conserved cysteines 8 and 108 appear important for inflammasome activation by sterile, but not infectious insults. Our results define common and agonist-specific regions of NLRP3 that likely mediate ligand-specific responses, discount the hypothesis that NLRP3 inflammasome activation has a unified mechanism, and implicate NLRP3 as an integrator of agonist-specific, inflammasome activating signals.

## Introduction

The pleiotropic cytokine IL-1β, a master regulator of inflammation, is essential for controlling infection, maintaining host homeostasis, and supporting adaptive immunity (1). IL-1β defends against infection by rapidly recruiting neutrophils to the infected site, activating endothelial adhesion molecules, driving production of anti-microbial peptides, and the elaboration of other cytokines and chemokines. IL-1β also promotes Th17-type adaptive immune responses (1–6). Therapeutic blockade of IL-1β ameliorates the symptoms of several autoinflammatory diseases and promotes resolution (3, 7–10). IL-1β is synthesized as an inactive precursor and typically activated by caspase-1 cleavage via inflammasomes, multi-protein complexes formed after activation of certain intracellular receptors, most notably Nod-like receptors (NLRs) (11–16). Although macrophages are the best studied cellular source of IL-1β, inflammasomes along with their initiating NLRs are not restricted to macrophages and have been described in T cells as well as various epithelial cell lineages (17–19).

Among NLRs, NLRP3 is the most well-studied (12–16). NLR responses are largely agonist specific (e.g., NLRP1, anthrax lethal factor; NLRC4, bacterial flagellin) (20–22). However, the NLRP3 inflammasome is activated by structurally and chemically diverse triggers of human, microbial, and environmental origin (12, 13, 15, 23–37). Further, activating mutations in the NLRP3 gene mediate hereditary autoinflammatory diseases ranging in severity from the mild familial cold autoinflammatory syndrome (FCAS) to the severe neonatal-onset multisystem inflammatory disease (NOMID) (38–47). Moreover, dysregulated NLRP3 inflammasome responses are a contributing factor in various inflammatory and autoimmune diseases (41–43, 46, 47).

Knowledge of the mechanism(s) responsible for activation of the NLRP3 inflammasome is limited. Indeed, the sequence of events believed to be fundamental has not changed since the first descriptions of the inflammasome complex. The NBD-WD40 repeat-containing protein APAF-1 is structurally similar to NLRs and provided an initial model informing early inflammasome studies (48–51). APAF-1 assumes an at rest, inhibited conformation relieved by binding of cytochrome C from damaged mitochondria and dATP which facilitate APAF-1 oligomerization and assembly of the active apoptosome (52). By analogy, In the absence of an NLRP3 inflammasome agonist, NLRP3 is thought to be auto-repressed through interaction of the nucleotide-binding domain (NBD) with the LRRs (15). Recognition of various agonists by NLRP3 is thought to relieve this auto-repression allowing ATP-dependent oligomerization of NBD domains and exposure of the N-terminal Pyrin domain (PYD) which then recruits ASC and pro-Caspase-1 (53). Analogous to plant R proteins and Toll-like receptors (TLRs), the LRR domain of NLRs are also thought to be the receptor domain for various ligands (54). Although direct interaction of activating ligands with LRRs is widely attested for NLRP3 in the literature (12, 15), little evidence supports this hypothesis. In contrast, a recent study suggests that LRRs are dispensable for mouse Nlrp3 inflammasome activation (55).

Multiple mechanisms have been proposed to delineate NLRP3 inflammasome activation, but each has limiting caveats. First, activation by ATP requires K^+^ efflux mediated by the ATP-gated K+ P_2_X_7_R channel and is thought to involve the Pannexin1 hemi-channel and influx of agonists (12, 56, 57). However, while Pannexin1 inhibitors block NLRP3 activation of caspase-1, this response is unimpeded in Pannexin 1-deficient macrophages (58). Further, certain NLRP3 triggers including particulate agonists are unable to enter the cytoplasm through plasma membrane pores (59, 60). Secondly, phagosome destabilization and rupture during phagocytosis of various particles releases cathepsin B, a protease initially proposed to activate NLRP3 (29, 61). However, although impaired by cathepsin B inhibitors (29, 61, 62), NLRP3 inflammasome activation by cathepsin B deficient macrophages is unaffected (62). Finally, reactive oxygen species (ROS) generated either via NADPH oxidase or mitochondrial damage are thought to potentiate NLRP3 inflammasome activation by facilitating NLRP3 association with thioredoxin interacting protein (TXNIP) (63, 64). Most NLRP3 agonists have been proposed to generate ROS, thus providing a unifying convergent activation mechanism for the various agonists (61). However, some NLRP3 agonists may not induce ROS and certain means of ROS induction fail to activate the NLRP3 inflammasome (16). Moreover, caspase-1 activation is not completely dependent upon TXNIP (65). Some studies suggest that TXNIP is not complexed with activated NLRP3 and may inhibit NLRP3 activation in mouse Kupfer cells (65, 66). In contrast, ROS may lead to nitrosylation-dependent degradation of NLRP3 and caspase-1 (67–70), or only be required for the “priming” first signal that induces NLRP3 expression prior to NLRP3-mediated inflammasome activation (29).

Given the diversity of agonists along with the above caveats and contradictory features of the published data, the molecular basis for NLRP3 inflammasome activation is, at best, incompletely understood. As this mechanism, or mechanisms, is still unresolved, global blockade of IL-1β, independent of specific inflammasomes, remains the preferred treatment modality for inflammasomopathies despite patient susceptibility to life-threatening infections (67, 69, 71–73). Better understanding of NLRP3 inflammasome activation will facilitate development of safer therapies that selectively inhibit NLRP3.

In this study we sought to establish the structural elements of NLRP3 required to respond to various activating agonists. Using truncated and chimeric forms of NLRP3, we find that several long-held paradigms concerning NLRP3 inflammasome activation are largely incorrect. We propose a new paradigm where NLRP3 serves as a signal-integrating scaffold with distinct responsive elements within the N-terminal, nucleotide-binding, and LRR domains that facilitate and potentially regulate agonist-specific inflammasome activation. Moreover, mutational analysis of N-terminal cysteines 8 and 108 demonstrate that these conserved residues may facilitate responses to sterile, but not infectious agonists.

## Materials and Methods

### Cell Culture

HEK293T cells were cultured in complete DMEM media (10% FBS, 1% L-glutamine, and 0.1% penicillin/Streptomycin) at 37°C, 5% CO_2_. Cells were maintained at sub-confluence and split every 2–3 days. Cell numbers and viability were determined by trypan blue exclusion.

### Bacterial Culture

*Francisella novicida* U112 colonies were isolated on modified Mueller-Hinton (MH) agar plates and cultured in modified MH broth (Difco) with ferric pyrophosphate and IsoVitalex (BD Biosciences) (74). *Listeria monocytogenes* was cultured in Brain Heart Infusion broth. Cultured bacteria were aliquoted and stored frozen at −80°C. Viable bacterial numbers (cfu) in frozen vials were calculated by plating of serially-diluted cultures method. At the time of infection, frozen vials of bacteria were thawed and used for infection according to the indicated MOI.

### Expression Constructs and Cloning

Expression plasmids encoding human NLRP1, NLRP2, caspase-1, and pro-IL-1β were all obtained from Open BioSystems. Human FLAG-NLRP3, and myc-ASC have been described previously (47). All restriction enzymes were obtained from New England Biolabs. Oligonucleotide primers used in this project were produced by Integrated DNA Technology and are listed in (Table 1). Truncation mutants of NLRP3 at 134, 432, 532, and 797 were generated using QuickChange (Applied BioSystems) to introduce stop codons. The Walker-A mutant was generated as described previously (66) using NLRP3 1–432 as template DNA. For chimeric NLRP332, the NLRP3 LRR residues 717–1034 were substituted for the corresponding LRR of NLRP2 (residues 790–1062). A BamHI site was introduced by QuikChange into pcDNA3-FLAG-NLRP3 at residue 716 and the NLRP3 LRRs were excised via BamHI/XhoI restriction digest. The LRRs of NLRP2 were PCR amplified with primers containing restriction enzyme sites for BamHI and XhoI. BamH1/XhoI digested PCR products were ligated into the BamHI/XhoI cut vector with T4 ligase. For PYD constructs, the PYD of NLRP1 (12–102) (synthesized by Genewiz) or NLRP2 (aa 2–132) introduced into pcDNA3-FLAG-NLRP3ΔPYD (NLRP3 residues 12–101) using KpnI sites to generate the chimeras NLRP133 and NLRP233, respectively. All constructs were sequenced to ensure the absence of unintended mutations.

**Table 1:** Primers Used in the Study.

**Primer name (purpose)**	**Sequence**
hNLRP2 (Add new EcoRI site and frame-correction)	F: 5′- CCACGTGGGACAAGAATTCATGGTGTCTTCGGC-3′
	R: 5′- GCCGAAGACACCATGAATTCTTGTCCCACGTGG-3′
hNLRP2-EcoRI (Insert EcoRI at the end PYD of pcDNA3FLAG-hNLRP2)	F: 5′-GCTTTGAAATCCTTGAATTCAAGGAAGCCTCTATC-3′
	R:5′-GATAGAGGCTTCCTTGAATTCAAGGATTTCAAAGC-3′
hNLRP332 (Amplify LRRs; 7901062 from NLRP2 with BamHI and XhoI sites to swap with LRRs of NLRP3;718-1034)	F: 5′-GGATCCTTGGTGTCTTGTTCCGCTAC-3′
	R: 5′-CTCGAGTCAGATCATGAAGTCATGAGAAG-3′
hNLRP3 1-797 (introduce a stop codon at aa 797)	F: 5′-CAGCAGCAACCAGAAGTAGGTGGAGCTGGACCTG-3′
	R: 5′-CAGGTCCAGCTCCACCTACTTCTGGTTGCTGCTG-3′
hNLRP3 1-532 (introduce a stop codon at aa 532)	F: 5′-CTTTGCCGCCATGTACTACTAGCTGGAAGAGGAAAAGG-3′
	R: 5′-CCTTTTCCTCTTCCAGCTAGTAGTACATGGCGGCAAGG-3′
hNLRP3 1-432 (introduce a stop codon at aa 432)	F: 5′-CAAGAGCCTTGCCTAGA CATCCAAGACCACCACCGCGG TGTACG-3′
	R: 5′-CTGGACTGAAACAGCAGATGGAGAGTGGCAAGAGCCTTG CCTAGACATC-3′
hNLRP3 1-134 (introduce a stop codon at aa 134)	F: 5′-TCCATATGTAAAATGAAGAAATGATTACCGTAAGAAGTAC-3′
	R: 5′-GTACTTCTTACGGTAATCATTTCTTCATTTTACATATGGA-3′
hNLRP233 (Amplify hNLRP2 PYD with KpnI site; allows NLRP2-PYD insertion in NLRP3ΔPYD)	F: 5′-GGTACCTGGAGCAGCTCAGCCAGG-3′ R: 5′-GGTACCTTTATTAAAGGATTTCAAAGCTGC-3′
hNLRP133 (Amplify hNLRP1 PYD with KpnI site; allows NLRP1-PYD insertion in NLRP3ΔPYD)	F: 5′-GGTACCTGGAGTTCCTGAAGAAGGAG-3′ R: 5′-CATTCCCCTACAGCCCAAGGTACC-3′
hNLRP3C8S/A (Mutate C8 to S, A, or G)	F: 5′-AAAATGGCAAGCACCCGCKSCAAGCTGGCCAGGTACCTG-3′
	R: 5′-CAGGTACCTGGCCAGCTTGSMGCGGGTGCTTGCCATTTT-3′
hNLRP3C38S/A (Mutate C8 to S, A, or G)	F: 5′-ATCCTCCCCAGAAGGGCKSCATCCCCCTCCCGAGGGG-3′
	R: 5′-CCCCTCGGGAGGGGGATGSMGCCCTTCTGGGGAGGAT-3′
hNLRP3C108S/A (Mutate C8 to S, A, or G)	F: 5′-GAATCCCACTGTGATAKSCCAGGAAGACAGCATTG-3′
	R: 5′-CAATGCTGTCTTGGSMTATCACAGTGGCATTC-3′
pCX4-Puro-NLRP3 (Retroviral cloning of NLRP3 and mutants)	F:5′-CCATCCTCTAGACTGCCGGATCCATGGACTACAAGGACGATGACG-3′
	R: CCGCACGCGTCGGTCCGGAATTCCTACCAAGAAGGCTCAAAGACG-3′

### Inflammasome Reconstitution and Activation

Inflammasome reconstitution in HEK293T cells which lack NLRP3, ASC, Caspase-1, and most pathogen receptors, allows evaluation of specific receptors by agonists that activate multiple pathways. In addition, this system also avoids significant caveats present for tractable macrophage systems including transducible immortalized mouse macrophages. Specifically, assumptions that human and mouse NLRP3 are functional equivalents and artifacts related to cell death. In our hands, these cells, exhibit minimal cell death (<10%) at the agonist concentrations used. Further, the human IL-1 ELISA used does not detect unprocessed IL-1β released by dead/damaged cells.

Transfections were performed using 2.5 μl of FuGENE 6 per μg of DNA. For inflammasome reconstitution, HEK293T cells were seeded (2.5 × 10^4^ cells/well/ml) in 24-well plates. After overnight culture, cells were transfected with pro-caspase1 (40 ng), pro-IL1β (200 ng), and ASC (8 ng) with an NLRP or empty vector pcDNA3 (100 ng) plasmid. For *Francisella novicida* infection, at 4 h post-transfection, cells were infected with U112 (100 MOI). After 24 h, culture supernatants were collected by centrifugation. For treatment with H_2_O_2_ or nigericin, and for *L. monocytogenes* infection, 18 h post-transfection, cells were treated with H_2_O_2_ (100 μM/well) for 1 h, with nigericin (5 μm/well) for 2 h, or infected with *L. monocytogenes* (20 MOI) and incubated 6 h. Culture supernatants were collected and IL-1β measured by ELISA (Life Technologies) per the manufacturer's instructions.

### Western Blotting

Cells were lysed in lysis buffer (50 mM Tris-HCl (pH 7.4), 150 mM NaCl, 1% Nonidet P-40 (v/v), 2 mM EDTA, 2 mM DTT with protease inhibitors). Protein concentrations were normalized by performing a protein estimation assay by BCA and 10 or 20 μg of protein was subjected to SDSPAGE (10% or 4–20%) and transferred to 0.2 μm PVDF, 1 h at 130 volts. After blocking with 5% milk in TTBS-(1X Tris-buffered saline (TBS), 0.05% Tween-20), the membrane was probed with anti-FLAG antibody (Clone M2; Sigma Aldrich) (1/1000). Bound FLAG antibody was detected with goat anti-mouse IgG-HRP (1/2500) using SuperSignal West Dura HRP detection reagents and visualized using an Alphaimager chemiluminescence system (Alpha Innotech).

### Speck Assay

HEK293T cells were seeded (5 × 10^5^) cells/well/ml in 6-well plates with coverslips. After overnight culture, cells were transfected with 1 μg of myc-ASC and NLRP3 (Full length or 1–93). 18 h post-transfection, cells were washed three times with 1X PBS. Cells were fixed with 4% paraformaldehyde (PFA) for 15 min at room temperature and permeabilized with 0.1% TritonX-100 for 10 min at room temperature. Fixed cells were blocked in PBS containing 5% fish gelatin, 1% BSA, 0.05% Triton X-100 for 1 h at room temperature. After blocking, cells were stained with mouse anti-FLAG antibody (Clone M2; Sigma Aldrich) (1/1000) and rabbit anti-ASC (N15)-R (Santa Cruz) (1:1000) in wash buffer (PBS containing 1% fish gelatin, 1% BSA, and 0.5% Triton X-100) for 2 h. Cells were washed three times with wash buffer, followed by incubation with Alexa Fluor^®^488 goat-antimouse IgG (1:1000) or Alexa Fluor^®^594 goat-anti-rabbit IgG2a (1:1000) in wash buffer for 1 h.

### Homology Modeling

Amino acid sequences of NLRPs LRRs and PYD were used to develop homology models using SWISS-MODEL (https://swissmodel.expasy.org/). The template was selected on a strict criterion of >30% sequence identity and >80% target coverage. Template characteristic details are provided in Figure S1. The homology models were aligned to NLRP3 LRRs using PyMOL and calculated rms value was tabulated (Figure S1). The crystal structure for NLRP1 LRRs was available (PDB Id: 4im6.1A). Crystal structure of PYD of NLRP1 & 3 were available (PDB Id: NLRP3- 3qf2; NLRP1- 1pn5).

### Expression of hNLRP3 in Immortalized Mouse Macrophages

Immortalized macrophages (iMCs) were kind gift from Dr. Douglas Golenbock lab (UMass Medical School, Worcester) and were generated using J2 recombinant retrovirus carrying v-myc and v-raf oncogenes as described (61, 75). The full-length and truncated mutants of hNLRP3 and were cloned in retroviral transfer plasmid pCX4 Puro using SLIC protocol as described (76, 77). HEK293T cells were transfected with the MLV gag-pol, VSVG, and pCX4-NLRP3 (FL) or pCX4NLRP3-1-432 or pCX4-NLRP3-1-134, using GeneJuice transfection reagent (EMD Millipore) following the manufacturer's recommendations. For the virus production, culture supernatant was collected 24 h post-transfection and filtered using a 0.45 μm pore filter. Fresh media was added to the cells and was harvested using the same protocol after 24 h and stored at −80°C until use. iMCs were cultured in filtered supernatant containing virus particle (24 h media) and supplemented with 8 μg/ml Polybrene for 24 h. After 24 h, transduced cells were selected with 5 μg/ml of puromycin for 6 days. The transduced cells were passaged for three generations in media containing 3 μg/ml of puromycin.

### Time of Flight Speck Assay

HEK293T cells (2 × 10^5^) were seeded in 12 well plates in 1ml DMEM and incubated overnight at 37°C with 5% CO_2_. Individual wells were transfected with plasmids encoding GFP-ASC (50 ng) with NLRP3 (WT or mutants) (100 ng) and incubated at 37°C with 5% CO_2_. 4 h post-transfection, cells were infected with *Francisella novicida* U112 or left uninfected for 24 h. After 24 h, cells were treated with 50 μl trypsin-EDTA (Corning; Cat.#25-053-Cl) per well and fixed with 4% paraformaldehyde (PFA) (EMS; Cat.#15710) for 15 min at room temperature. Cells were washed once with 1X PBS and resuspended in 1X PBS supplemented with 0.5 mM EDTA. The samples were acquired on LSRII flow cytometer equipped with 405, 488, and 642 nm lasers with long-pass filter of 505 nm and band-pass filters of 450/50, 530/30, and 660/20 nm. Acquisition was done using BD DIVAS software. Data was analyzed using FlowJo. Samples were gated to exclude debris and cell doublets. Singlet population was further gated for GFP staining. A stop gate of 10^4^ cells was set on the GFP-positive gate. The percentage of cell containing ASC specks was determined by analyzing the height (H), width (W), and area (A) of the GFP pulse area (high H:A and low W:A indicates speck positive cells) as described previously (78).

### Statistical Analysis

At least three independent experiments were performed with two to three technical repeats. Twoway ANOVA with Dunnet, Sidak, or Tukey multiple comparison test was used to compare means as indicated. *p*-values of ≤ 0.05 were considered statistically significant. All statistical analysis was performed using GraphPad Prism6 software.

## Results

### The LRRs of NLRP3 Are Not Required for Inflammasome Activation

**Figure 1:**
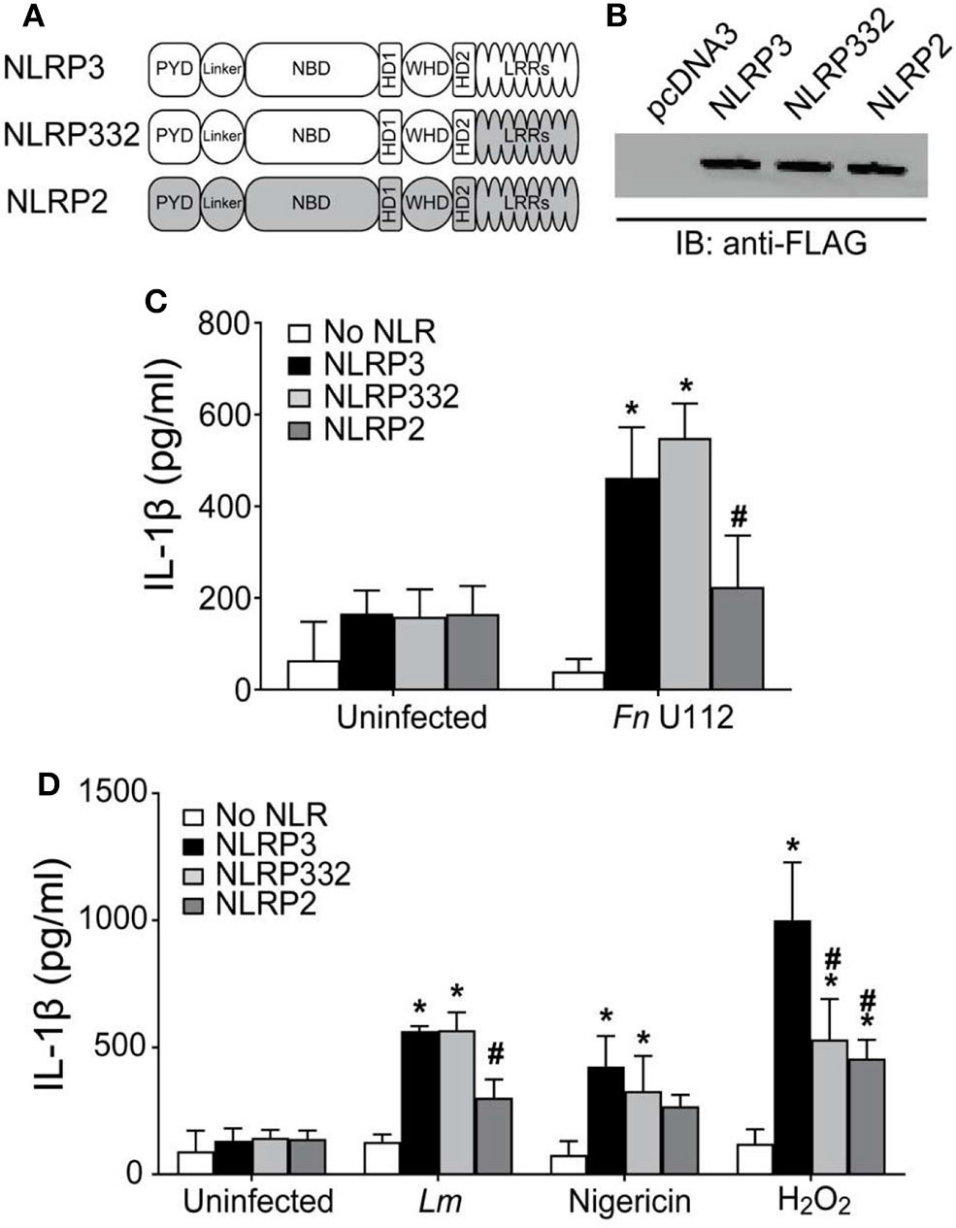
The LRRs of NLRP3 are dispensable for inflammasome activation. **(A)** Schematic representation of the LRR domain substitution construct. **(B)** Representative western blot of FLAG-tagged NLRs from HEK293T cell lysate. 10 μg total protein was loaded in each well. **(C,D)** IL-1β response of HEK293T cells expressing ASC, pro-Caspase-1, pro-IL-1β, and NLRP3, NLRP2, or the LRR domain substitution chimera NLRP332. **(C)** At 4-h post-transfection, cells were infected with Fn U112 (MOI 100). **(D)** 18–20 h post-transfection, cells were infected with *Listeria monocytogenes* (Lm) (MOI 20; 6 h) or treated with 100 μM H_2_O_2_ (1 h) or 5 μM nigericin (2 h). IL-1β in culture supernatant was measured by ELISA. Data represent means ± SEM for a minimum of three independent experiments. ^*^p < 0.0001 for comparison with respective untreated controls; ^#^*p* < 0.0001, two-way ANOVA followed by Tukey (^*^) and Sidak's (^#^) multiple comparison test.

NLRP3 inflammasome agonists are structurally diverse, but while the NLRP3 LRRs are thought to be involved, how NLRP3 specifically recognizes agonists is unclear. The NLRP3 LRRs are encoded by exons 4–9 and comprise 11 repeats of the leucine-rich repeat consensus sequence (79). However, whether the LRRs confer specificity for any agonist is also unclear. Pathogen specificity of the plant NLR L5 was demonstrated by substituting LRR domains of L5 with L6 isoform (12), indicating the utility of a substitution approach to reveal LRR specificity. Recently, partial LRR truncations of mNlrp3 were reported to be non-responsive to nigericin stimulation (55). However, the reason for this result is unclear as shorter NLRP3 constructs were responsive and only sterile agonists and nigericin were examined. To better establish whether the NLRP3 LRRs are critical for sensing particular agonists, we generated an LRR-domain chimeric mutant, “NLRP332” by substituting the LRR of NLRP2 for the corresponding domain of NLRP3 (Figure 1A). The corresponding LRRs of NLRP2 were determined to be the most homologous to the NLRP3 LRRs using both modeling and structural alignment analyses (sequence identity ≥30% with target coverage ≥80% and low atomic model rms score) (Figure S1). Expression of NLRP332 was comparable to that of wild-type NLRP3 (Figure 1B), thus this substitution does not alter expression of the chimeric mutant. As *Francisella novicida* U112 (Fn U112) does not activate the NLRP2 inflammasome (80), we tested whether FnU112 infection activates an NLRP332 inflammasome using the well-established inflammasome reconstitution assay (11, 41). Inflammasome reconstitution in HEK293T reproduces results from inflammasome activation in macrophages and has been frequently used to characterize NLRP3 inflammasome activation by various agonists (81). Moreover, as HEK293T cells do not express any NLR, ASC, or Caspase-1, NLRP3 inflammasome function can be studied in isolation using these cells without cross-talk from other NLRs (41, 82–84). As we previously demonstrated (83), Fn U112 infection of NLRP3 inflammasome-reconstituted HEK293T cells elicited robust IL-1β production and cells lacking NLRP3, or expressing NLRP2, yielded IL-1β levels comparable to uninfected controls (Figure 1C). However, the IL-1β response of cells expressing NLRP332 was comparable to that of wildtype NLRP3. Thus, the NLRP2 LRRs may substitute for those of NLRP3 with respect to sensing of Fn U112. Alternatively, the NLRP3 LRR domain may not be required to sense this agonist.

The response of NLRP332 to *L. monocytogenes* (Lm) infection and stimulation with the sterile NLRP3 inflammasome agonists nigericin and H_2_O_2_ was also examined (Figure 1D). As with Fn U112, NLRP332, and NLRP3 inflammasome activation was comparable after Lm infection or nigericin stimulation. However, stimulation with H_2_O_2_ only partially activated the NLRP332 inflammasome (~50% vs. NLRP3), indicating that the NLRP2 LRRs cannot completely substitute for those of NLRP3 and suggesting that some feature of NLRP3's LRRs is required to respond to H_2_O_2_. Even though the NLRP3 LRRs may sense H_2_O_2_, the LRRs of NLRP2 preserve NLRP3 function for all the other NLRP3 inflammasome agonists tested. Interestingly, although thought to be NLRP3-specific, H_2_O_2_ also activated the NLRP2 inflammasome. Further, NLRP2 and NLRP332 responses to H_2_O_2_ were comparable. Whether sensing of H_2_O_2_ by the NLRP2 LRRs might account the partial activation of NLRP332 is unclear. However, as the LRRs of NLRP2 and NLRP3 share similar structures but lack highly homologous primary sequences, NLRP2 LRR sensing of H_2_O_2_ seem less likely (79). Thus, similar to mouse NLRP3, the LRRs of human NLRP3 might not be as important for agonist sensing as previously believed.

### Distinct LRR and LRR-Like/HD2 Structures Modulate Agonist-Specifc NLRP3 Activation

**Figure 2:**
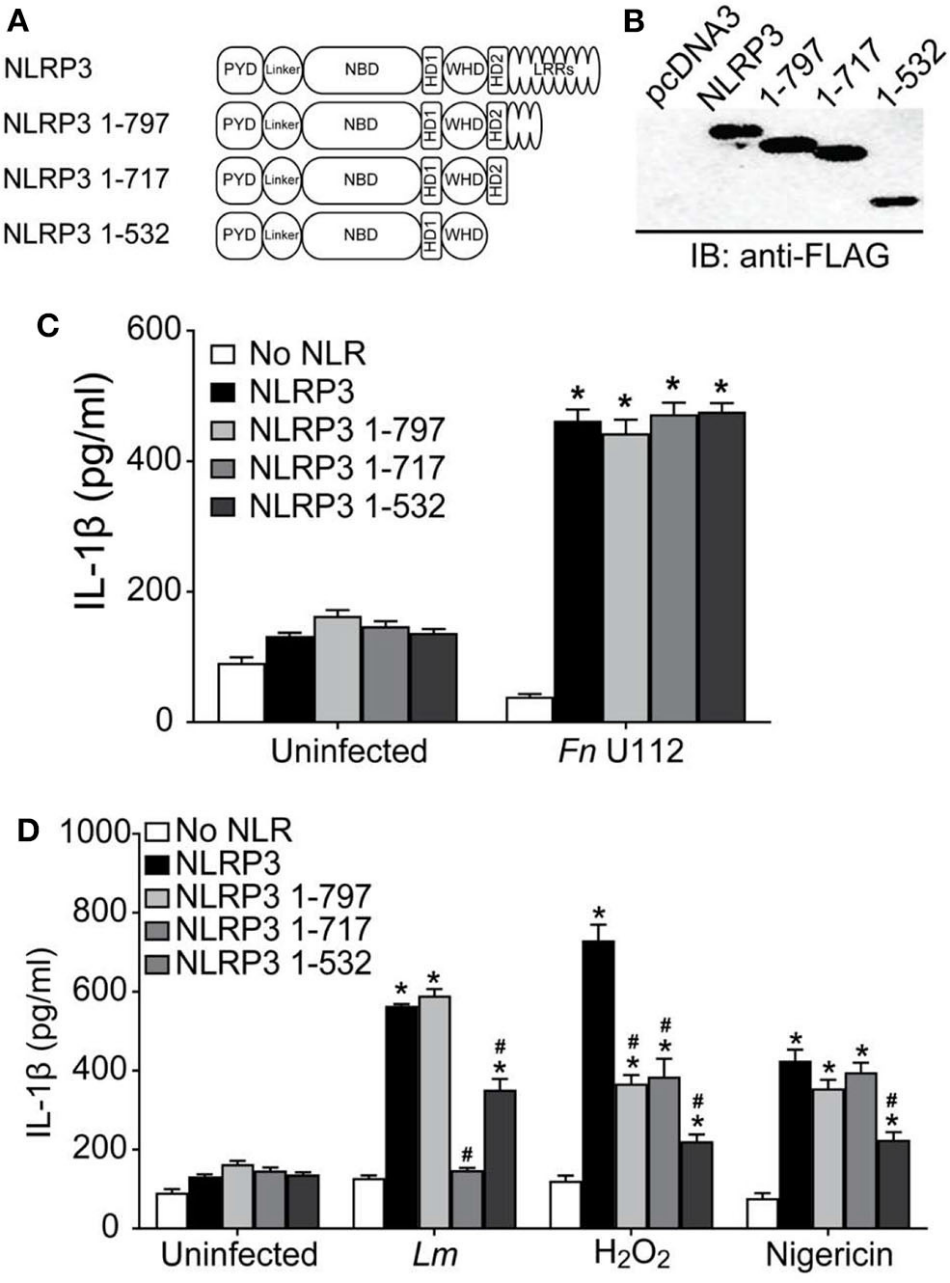
NLRP3 activation by diverse agonists requires distinct protein structures. **(A)** Schematic representation of C-terminal LRR domain truncation mutants. **(B)** Representative western blot of FLAG-tagged NLRP3 and its C-terminal LRR truncation mutants from HEK293T cell lysate. 10 μg total protein was loaded in each well. EV-Empty vector. **(C,D)** HEK293T cells expressing ASC, pro-Caspase-1, pro-IL-1β, and NLRP3, or the indicated LRR domain truncation mutants. **(C)** At 4-h post-transfection, cells were infected with Fn U112 (MOI 100). **(D)** 18–20 h post-transfection, cells were infected with *Listeria monocytogenes (Lm)* (MOI 20; 6 h) or treated with 100 μM H_2_O_2_ (1 h) or 5 μM nigericin (2 h). IL-1β in culture supernatant was measured by ELISA. Data represent means ± SEM for a minimum of three independent experiments. ^*^p < 0.0001 for comparison with respective untreated controls; ^#^*p* < 0.0001, two-way ANOVA followed by Tukey's (^*^) and Sidak's (^#^) multiple comparison tests.

To further examine the requirement for the NLRP3 LRRs, a series of C-terminal NLRP3 truncation mutants were constructed (Figure 2A) and tested in inflammasome reconstitution assays. We also deleted LRR-like sequences (also called helical domain 2 (HD2)) (84, 85) present in the Cterminal end of exon 3 which encodes the NACHT domain 3 (79). Expression of each truncation mutants was comparable to full-length NLRP3 (Figure 2B). Surprisingly, all the truncation mutants exhibited a full response to Fn U112 indistinguishable from that of wt NLRP3 (Figure 2C). Thus, truncation does not generally disrupt the structure and function of NLRP3. The LRRs (718–1,036) and LRR-like sequences/HD2 (532–717) of NLRP3 were completely dispensable for inflammasome activation by Fn U112. The LRRs and LRR-like sequences therefore do not act as a critical ligand sensor for this pathogen. Thus, the responsive elements for detection of Fn U112 are present in the N-terminal 532 residues. Further, there was no significant difference in IL-1β production between unstimulated cells expressing NLRP3 and those expressing any of the truncations (Figures 2C,D). Thus, and in confirmation of recently published work, NLRP3 LRRs do not maintain an inactive, auto-repressed NLRP3 conformation as previously suggested.

As the LRRs do not appear to be a direct or indirect sensor for Fn U112, sensing of some agonists must require NLR structure features distinct from the LRRs, as seen with some plant NLRs (83). However, such a mechanism would not exclude LRR sensing of other agonists. To determine whether NLRP3's LRRs are equally dispensable for other bacterial and sterile stimuli, Lm and H_2_O_2_ were used. NLRP3 inflammasome activity following Lm infection was unaffected by removal of amino acids 798–1,036 (NLRP3 1–797) but was completely abolished when residues 718–797 were removed (Figure 2D). Surprisingly, further deletion of residues 533–717 (NLRP3 1–532) significantly restored the response (>50% of wildtype). Thus, NLRP3 residues 1–532 suffice for partial activation by Lm infection and contain responsive elements. Further, amino acids 533–717 (LRR-like sequences/HD2) contain a negative regulatory element preventing activation during Lm infection. However, the LRRs within 718–797 contain a positive regulatory element. In contrast to both bacterial stimuli, H_2_O_2_-stimulation of NLRP3 1–797 reduced inflammasome activation to nearly 50% of full-length (Figure 2D). However, removal of residues 718–797 had no further impact. H_2_O_2_-elicited inflammasome activity was further decreased for NLRP3 1–532, although IL-1β production was still significantly higher than background levels. Thus, both the most distal LRRs (797–1,036) and the LRR-like sequences/HD2 (532–717) are likely positive regulatory elements for NLRP3 inflammasome activation by H_2_O_2_ and are likely involved in direct or indirect sensing of this stimuli_._

Immortalized BMDMs expressing mouse NLRP3 1–794 (comparable to our 1–797 truncation) were fully responsive to stimulation with nigericin, SiO_2_, and alum (55) a result quite different from data with Fn U112, Lm, and H_2_O_2_ above. In our hands, removal of NLRP3 residues 717–1,036 had little impact on the inflammasome response to nigericin (Figure 2D). However, while the response of NLRP3 1–532 to nigericin was reduced, significant inflammasome activation was still evident.

These results are comparable to those reported using similar mouse Nlrp3 constructs (1–794, 1,731, and 1–541) in BMDMs (55) Thus, our results confirm that the C-terminal, canonical LRRs of human NLRP3, as with mouse Nlrp3, are dispensable for inflammasome activation by nigericin and confirm that inflammasome responses in reconstituted HEK293T cells are comparable to those with mNlrp3 using immortalized macrophages. Moreover, residues 532–717 appear to contain a positive regulatory element for activation by nigericin indicating that the LRR-like sequences may serve as a potential sensor for nigericin.

### NLRP3 1–134 Is Minimally Required for Agonist Specific Inflammasome Activation

**Figure 3:**
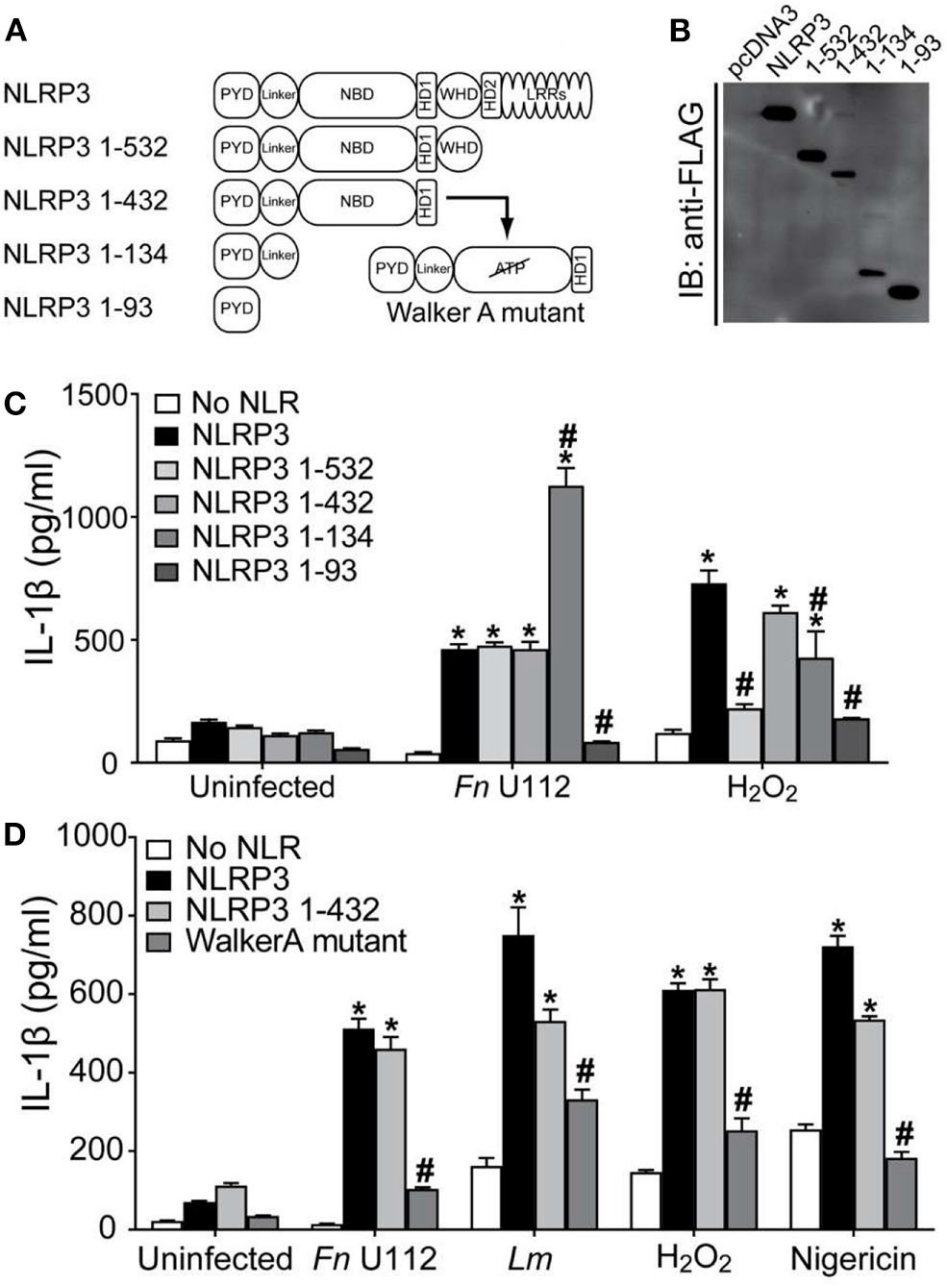
NLRP3 1-134 is minimally required for agonist specific inflammasome activation. **(A)** Schematic representation of NBD mutants. **(B)** Representative western blot of FLAG-tagged NLRP3 and NBD mutants from HEK293T cell lysate. 10 μg total protein was loaded in each well. EV-Empty vector. **(C)** IL-1β response of HEK293T cells expressing ASC, pro-Caspase-1, pro-IL-1β, and NLRP3, or the indicated truncation mutants. At 4-h post-transfection, cells were infected with Fn U112 (MOI 100) or 18–20 h post-transfection, cells were treated 100 μM H_2_O_2_ (1 h). **(D)** IL-1β response of HEK293T cells expressing ASC, pro-Caspase-1, pro-IL-1β, and NLRP3, or the indicated NBD mutants. For Fn U112 and H2O2, cells were treated as in **(C)**. At 18–20 h post-transfection, cells were infected with *L. monocytogenes* (Lm) (MOI 20; 6 h) or treated with 5 μM nigericin (2 h). IL-1β in culture supernatant was measured by ELISA. Data in **(C,D)** represent means ± SEM for a minimum of three independent experiments. ^*^p < 0.0001 for comparison with respective untreated controls; ^#^*p* < 0.0001, two-way ANOVA followed by Tukey's (^*^) and Sidak's (^#^) multiple comparison tests.

The first 686 residues of mouse Nlrp3 were recently reported to be minimally sufficient for inflammasome activation (55). However, unlike mouse Nlrp3 1–541, human NLRP3 1–532 is sufficient for at least partial inflammasome activation by all agonists tested. Thus, at least one agonist-responsive region is present within the N-terminal portion of NLRP3. Further, the minimal sequence required for agonist elicited human NLRP3 function is likely contained within 1–532, rather than residues 1–686 reported for mNlrp3. To refine the regions of NLRP3 sufficient for inflammasome activation by different agonists, additional NLRP3 truncations were generated (Figure 3A) and evaluated. NLRP3 1–432 retains the ATP/ATPase domain (133–432). NLRP3 1–134 deletes the nucleotide binding region but retains residues 1–93, the Pyrin domain which binds ASC, and amino acids 94–132, a region of unknown function encoded by exon 2. NLRP3 1–93 contains only the Pyrin domain.

Expression of NLRP3 1–432 and 1–134 was somewhat lower than that of full-length NLRP3 and the other constructs (Figure 3B). In the absence of agonist, none of these constructs exhibited a gain-of-function, while activation of NLRP3 1–432 with Fn U112 was indistinguishable from that of full-length NLRP3 and NLRP3 1–532 (Figure 3C), suggesting amino acids 433–532 are also not required for this activator. In sharp contrast, despite NLRP3 1–532 being almost completely unresponsive to H_2_O_2_, further deletion of 433–532 restored activation with H_2_O_2_ to near wildtype levels. Thus, residues 433–532 are likely to negatively regulate inflammasome activation by H_2_O_2_ but are dispensable for the Fn U112 response. Unexpectedly, further deletion to remove the ATPbinding site (NLRP3 1–134) increased the inflammasome response to Fn U112 by over 2-fold. However, while NLRP3 1–134 responded significantly to H_2_O_2_ stimulation, this response was diminished to ~50% of wildtype NLRP3 (Figure 3C). Despite containing the complete PYD domain, NLRP3 1–93 was insufficient to form an inflammasome in response to either stimuli. Thus, amino acids 1–134 comprise the minimal sequence necessary for both H_2_O_2_ and Fn U112 to activate the NLRP3 inflammasome. Further, as removal of amino acids 135–432 led to differential responses to Fn U112 (increased) and H_2_O_2_ (diminished), sequence elements between 135 and 432 including the ATP-binding site may constrain or enhance inflammasome activation in an agonist-specific fashion. Collectively, residues 1–134 contain features sufficient to assemble an NLRP3 inflammasome in response to Fn U112 and H_2_O_2_. This data contrasts with the prevalent idea that C-terminal portions of NLRP3 sense agonists and demonstrates that at least for these agonists, the minimally responsive portion of NLRP3 is much smaller than previously suggested.

The nucleotide binding domain (133–432) appears to negatively regulate the inflammasome response to Fn U112 while positively regulating the H_2_O_2_ response, suggesting an ATP-binding- or ATPase activity-dependent process. The Walker A motif (residues 226–233) of NLRP3 binds the terminal phosphate of ATP and mutating this motif diminishes NLRP3 inflammasome activity, presumably by impairing ASC recruitment (86). We reproduced this Walker A mutation within the NLRP3 1–432 truncation mutant (Figure 3A). As anticipated, the ATP binding mutation significantly and substantially reduced inflammasome activity in response to all treatments (Figure 3D). Curiously, inflammasome activation of the Walker A mutant by Lm and H_2_O_2_ were ~40–50% of wildtype NLRP3. Thus, NLRP3 1–432 is similarly responsive to the various agonists tested, demonstrating that this region senses these agonists and retains ATP-dependence, essential features of NLR function. Further, since the ATP-binding defective Walker A mutant diminished activation by all agonists tested, the ATP-bound state is likely not responsible for the gain of function seen with deletion of residues 135–432. Of note, Fn U112 did not effectively activate the Walker-A mutant, but NLRP3 1–134 was twice as active as wildtype despite lacking the entire nucleotide binding domain.

### NLRP3 Cysteines 8 and 108 Are Required for Response to Sterile Agonists

**Figure 4:**
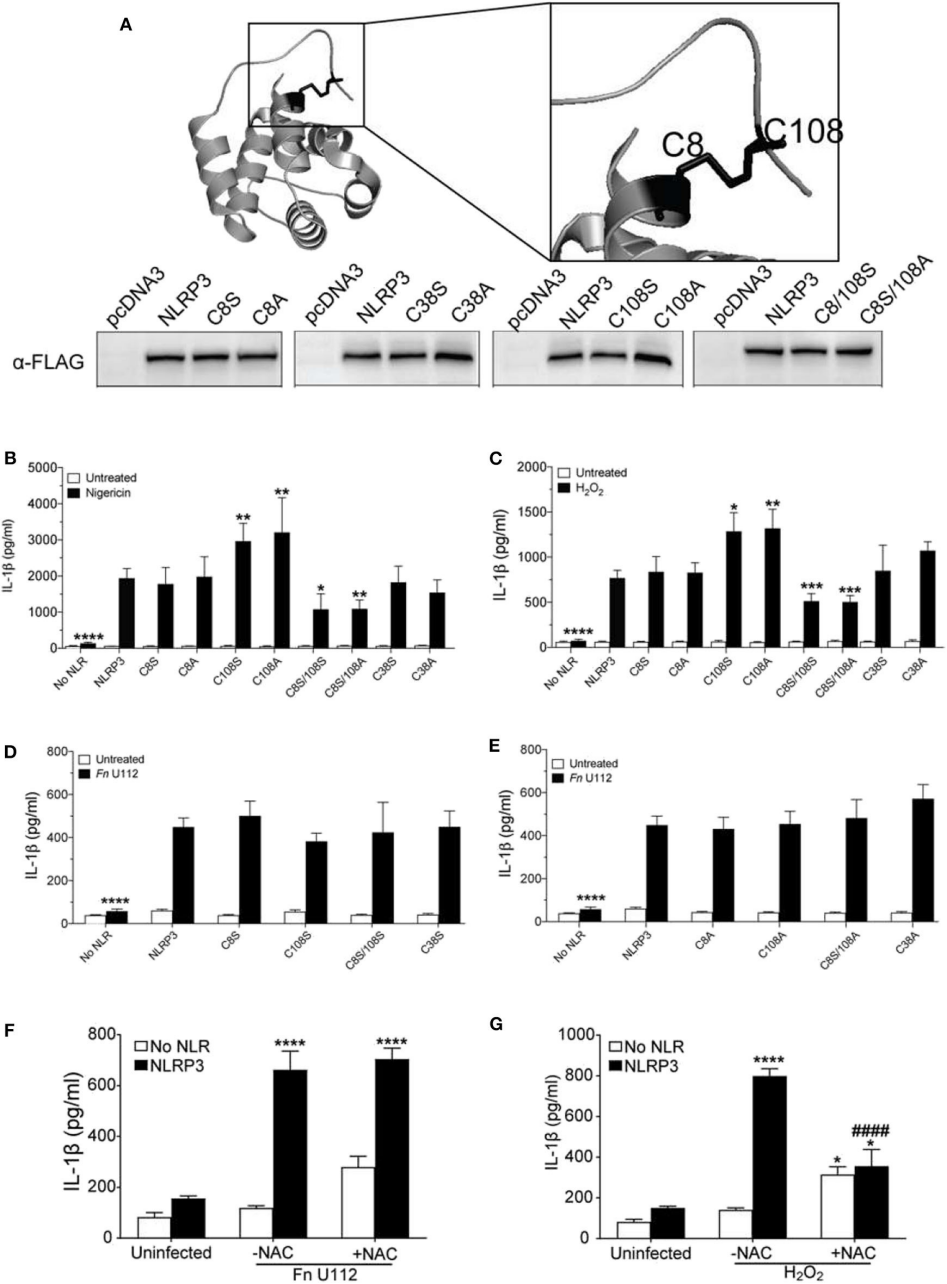
The NLRP3 response to sterile agonists requires conserved N-terminal cysteines. **(A)** Crystal structure of NLRP3-PYD (PDB ID:3QF2) generated with PyMOL showing positions of C8, C38, and C108 (top), Western blot of FLAG-tagged NLRP3 and its cysteines mutants from HEK293T cell lysate. 20 μg total protein was loaded (bottom). **(B,C)** IL-1β response of HEK293T cells expressing ASC, pro-Caspase-1, pro-IL-1β, and NLRP3, or C→S/A mutants. **(B)** At 18–20 h post-transfection, cells were treated 5 μM nigericin (2 h) or **(C)** with 100 μM H_2_O_2_ (1 h). **(D,E)** IL-1β response of HEK293T cells expressing ASC, pro-Caspase-1, pro-IL-1β, and NLRP3 or **(D)** C→S mutants or **(E)** C→A mutants. At 4-h post-transfection, cells were infected with Fn U112 (MOI 100) and IL-1β in culture supernatant was measured by ELISA. **(F)** IL-1β response of HEK293T cells as described in **(B)**. in the absence or presence of N-acetyl cysteine (NAC). IL-1β in culture supernatant was measured by ELISA. **(G)** IL-1β response of HEK293T cells expressing ASC, pro-Caspase-1, pro-IL-1β, and NLRP3 in the absence or presence of N-acetyl cysteine (NAC). **(F)** At 4-hr post-transfection, cells were infected with Fn U112 (MOI 100). **(G)** At 18–20 h post-transfection, cells were treated with 100 μM H_2_O_2_ (1 h). IL-1β in culture supernatant was measured by ELISA. Data in **(B)** through G represent means ± SEM for a minimum of three independent experiments. ^*^p < 0.05, ^**^p < 0.01, ^***^p < 0.001, and ^****^p < 0.0001 for comparison with respective untreated controls; ^####^*p* < 0.0001 for comparison with treated NLRP3, two-way ANOVA followed by Sidak's **(B–E)** and Dunnett's **(F,G)** multiple comparison tests.

While residues 1–134, encoded by exons 1 and 2, are sufficient for an NLRP3 inflammasome response to select agonists, the complete PYD domain (1–93) is not. Thus, both the Pyrin domain and the downstream residues 94–134 appear to cooperate to facilitate agonist sensing and functional inflammasome formation. The nature of this cooperation is unclear. Exon 1 encodes the well-defined PYD domain, while exon 2 encodes an unstructured linker of unknown function (94–134). Such linker regions, coded by single or multiple exons, are present in most NLRPs (Figure S2A). Residues 1–134, however, contain four cysteines at positions 8, 38, 108, and 130 that are specific to NLRP3 and highly conserved (Figures S2B,C). Moreover, crystallographic analysis of human NLRP3 amino acids 1–110 reveals that while C38 is a solvent exposed residue in the loop connecting helix 2 and 3, cysteines 8 and 108 are involved in a disulfide bond (87) (Figure 4A). This disulfide bond is proposed as a site where reactive oxygen species such as H_2_O_2_ might act to promote inflammasome activation. Post-translational oxidation states of cysteines frequently act as switches regulating protein function (88, 89), suggesting potential functions for these cysteines beyond a redox-elicited disulfide linkage. In contrast, individual mutations of the corresponding cysteines in mouse Nlrp3 (C6 and C104) do not reduce inflammasome activation by nigericin (55). To evaluate the contribution of these cysteines to human NLRP3 inflammasome function, cysteines to serine, and alanine mutants were generated.

Consistent with the prior study (90), neither mutation of cysteine 8 to serine (C8S) nor alanine (C8A) impaired the NLRP3 inflammasome response to nigericin and H_2_O_2_ (Figures 4B,C). Thus, the C8 sulfhydryl group appears dispensable for inflammasome function and the proposed C8–108 disulfide bond is not required for activity. Mutation of C108 to serine (C108S) or alanine (C108A) yielded a 1.5-fold increase in IL-1β production upon nigericin and H_2_O_2_ treatment (Figures 4B,C). Of note, the mouse NLRP3 C104A response is also higher than wildtype (55). Thus, the sulfhydryl group of C108 likely reduces nigericin and H_2_O_2_-mediated activation, implicating a regulatory function for C108 within the linker region (94–134). We also mutated the likely solvent exposed C38 within the PYD. Interestingly, NLRP3 inflammasome activation by H_2_O_2_, nigericin and Fn U112 was not altered by either C38A or C38S (Figures 4B–E). Thus, the cooperation between the Pyrin domain and the downstream residues 94–134 appear to be independent of a disulfide bond between C108 and either C8 or C38 of the PYD.

Unexpectedly, the inflammasome response to both nigericin and H_2_O_2_ was significantly impaired by mutation of both residues (C8/108S and C8A/108S). Thus, C8 and C108 may functionally complement each other, possibly through agonist-dependent oxidative modifications of the C8 and C108 sulfhydryl groups. Such modifications might further allow NLRP3 to discriminate between agonists. Consistent with this thinking, none of the cysteine mutants substantially impaired or improved the inflammasome response to Fn U112 (Figures 4D,E). Further, pretreatment with N-acetyl cysteine (NAC), an ROS scavenger, reduced the response to H_2_O_2_, but did not impair NLRP3 inflammasome activation by Fn U112 (Figures 4F,G). Thus, cellular oxidation status appears important for the response to the sterile agonist nigericin, but not for the bacterial agonist Fn U112. These results suggest that modification status of sulfhydryl groups at C8 and C108 might provide a molecular basis for distinguishing between sterile and bacterial NLRP3 agonists.

### The NLRP3 PYD Is Involved in Agonist Sensing

**Figure 5:**
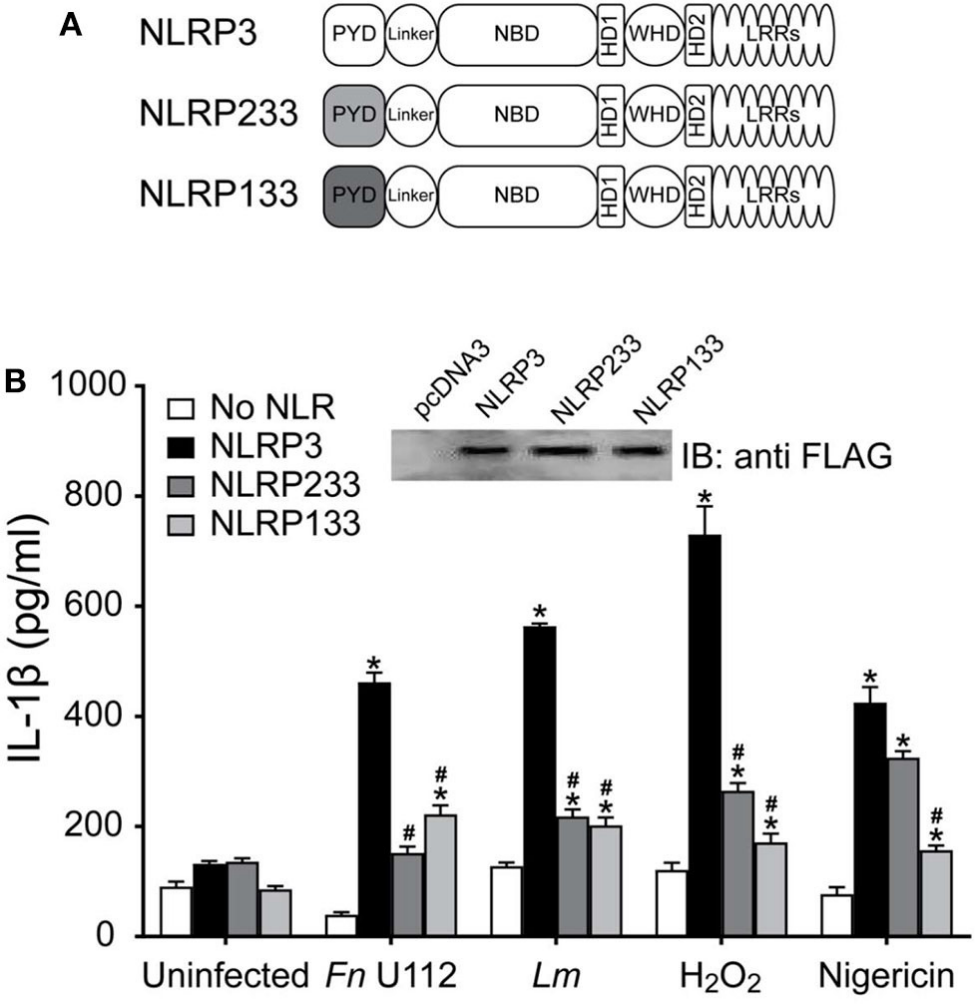
The PYD domain of NLRP3 is involved in agonist sensing. **(A)** Schematic representation of N-terminal PYD substitution mutants. **(B)** IL-1β response of HEK293T cells expressing ASC, pro-Caspase-1, pro-IL-1β, and NLRP3, or the indicated PYD substitution mutants stimulated by infection with Fn U112 (MOI 100) or *L. monocytogenes* (Lm) (MOI 20) or treated with 100 μM H_2_O_2_ or 5 μM nigericin. Supernatant IL-1β was measured by ELISA. Data represent the mean ± SEM for at least three independent experiments ^*^p < 0.0001 for comparison with respective untreated controls; ^#^*p* < 0.0001, twoway ANOVA followed by Dunnett's multiple comparison tests. (Inset) FLAG-tagged NLRP3 and N-terminal PYD substitution mutants expressed in HEK293T cells, 10 μg total protein was separated by SDS-PAGE (10%) and immunoblotted with anti-FLAG (M2).

The PYD (aa 1–93) is sufficient to mediate ASC association (Figure S2D) but is not sufficient for inflammasome formation (Figure 3C). However, functional coordination between the PYD and the exon 2 linker along with the selective requirement for C8 in the absence of C108 implicate the PYD as an agonist sensor. To better evaluate whether the NLRP3 PYD is involved in agonist sensing, a KpnI fragment containing residues 12–99 of NLRP3 was replaced with the corresponding residues from NLRP1 or NLRP2 to create PYD-chimeric NLRP3 mutants (Figure 5A). The PYD of NLRP1 and NLRP2 each interact with ASC to form inflammasomes (11, 41, 89, 91) and have ~40 and ~35% sequence similarity with NLRP3, respectively (Figure S3A). Expression of these chimeras was comparable to full-length NLRP3 (Figure 5B; inset). For all the agonists tested NLRP133 inflammasome activity was significantly lower than seen with NLRP3 (Figure 5B). Similarly, with NLRP233 IL-1β production was also much lower for all agonists except for nigericin which, surprisingly, elicited a robust response (Figure 5B). Thus, while the PYDs of NLRP1 and NLRP2 mediate inflammasome formation in their wildtype context, they cannot generally substitute for the PYD of NLRP3. Instead, a cognate pair consisting of the NLRP3 PYD and its corresponding linker appears essential for most agonists. The first seven amino acids of NLRP3 (MKMASTR), phosphorylation of serine 5, and cysteines 8 and 108 are important for the inflammasome response to nigericin (Figure 4B) (92, 93). These features are maintained in NLRP233 and NLRP133. Also, the PYD of NLRP2 has greater structural similarity to NLRP3 than NLRP1 (Figures S3B,C). Such features may help explain the selective responsiveness of NLRP233 to nigericin. However, elements within the PYD appear important for responding to NLRP3 agonists, a function that is likely distinct from binding ASC.

## Discussion

The paradigms that the NLRP3-LRRs serve to auto-repress inflammasome assembly and sense activating ligands directly or indirectly have been long-held. A recent truncation study of mouse Nlrp3 challenged these paradigms by demonstrating that the N-terminal 686 residues are sufficient for inflammasome assembly following nigericin, alum, and silicon dioxide stimulation (55). In our analysis of human NLRP3, LRR deletion did not result in spontaneous activation of the NLRP3 inflammasome, providing additional evidence that the LRR autorepression model is incorrect. However, our data reveals that structural features of NLRP3, including some LRRs, respond to or regulate NLRP3 inflammasome activation by distinct agonists. Further, our results may explain why some LRR truncations were non-responsive to nigericin in the mNLRP3 study. The observed diversity in the NLRP3 structures required for responsiveness to specific agonists demonstrates that the unified or convergent model of NLRP3 inflammasome activation by multiple agonists is almost certainly incorrect. Further, for some stimuli, the first 134 amino acids of NLRP3 may be sufficient to mediate inflammasome activation. Communication between the Pyrin domain and sequences linking it to the nucleotide binding domain, mediated in part by conserved cysteines, may help distinguish between sterile and non-sterile agonists.

While our data addresses the protein structural features of human NLRP3 (hNLRP3) required for inflammasome formation, whether these features differ between mouse Nlrp3 (mNlrp3) and hNLRP3 is incompletely understood. A recent study by Hafner-Bratkovic et al. examined activation of truncated mNlrp3 using reconstituted Nlrp3-deficient iMCs (55) and our results above using nigericin and crystal agonists with hNLRP3 are highly concordant. The same study also expressed hNLRP3 (1–667 and 1–688) in Nlrp3-deficient iBMDMs to recapitulate nigericin responsiveness of the corresponding mouse mutants (55) and the results suggested this system would be useful to evaluate our constructs. However, in our hands, while mNlrp3-sufficient cells produced IL-1β, mNlrp3-deficient iBMDMs expressing full-length hNLRP3 did not (Figure S4A). The reason for this disparity is unclear, however, it is most probable that hNLRP3 does not actually substitute for that of mouse. Indeed, in prior experiments hNLRP3 and hASC were both required to reconstitute NLRP3-deficient mouse macrophages (94). Similarly, Fn U112 activates the mNLRP3 inflammasome in inflammasome reconstituted cells (95), but mouse Nlrp3 does not substitute for human NLRP3 (Figure S4B). Why Hafner-Bratkovic et al. observed an hNLRP3 dependent IL-1β response in mouse cells is unclear, but extensive cell killing and release of intracellular (and likely unprocessed) proIL-1β suggests one possibility. Their stimulation of hNLRP3-iBMDMs was atypical (LPS for 11 h and 10 μM nigericin instead of 4 h and 5 μ M) and resulted in ~40–60% cell death with exceptionally high IL-1β (55). In our hands, these conditions elicited 20% less IL-1β than the more typical conditions from both wt and Nlrp3deficient iBMDMs, while hNLRP3 iMCs produced no IL-1β (Figure S4C) with as little as 10–30% cell death (Figure S4D).

Various stimuli activate the NLRP3 inflammasome including infectious bacteria [e.g., Francisella species (83, 96) and *Listeria monocytogenes* (97, 98) and sterile agonists such as nigericin, H_2_O_2_, and monosodium urate crystals (60, 63)]. Chimeric versions of NAIP2 and NAIP5 were used to demonstrate the importance of specific NAIP LRR sequences in the NLRC4 response to flagellin (99). Substituting NLRP3 LRRs with those from NLRP2 (NLRP332) did not impair inflammasome activation by Fn U112, Lm, or nigericin. However, H_2_O_2_ activation of NLRP332 was about 50% of NLRP3 and comparable to H_2_O_2_ activation of the NLRP2 inflammasome. Thus, the inflammasome response to H_2_O_2_ likely requires some feature within NLRP3's LRRs. Interestingly, although thought to be NLRP3-specific, H_2_O_2_ also activated the NLRP2 inflammasome. Further, NLRP2 and NLRP332 responses to H_2_O_2_ were comparable. Whether sensing of H_2_O_2_ by the NLRP2 LRRs might account the partial activation of NLRP332 is unclear. However, that the LRRs of NLRP2 and NLRP3 share similar structures but lack highly homologous primary sequences make this seem less likely (79). Even with the caveat that the NLRP3 LRRs may sense H_2_O_2_, the LRRs of NLRP2 preserve NLRP3 function for all the other NLRP3 inflammasome agonists tested here. The simplest explanation is that elements of the NLRP2 LRRs share agonist-sensing functions equivalent to those of NLRP3 but are insufficient for these agonists to activate NLRP2.

Deletion of LRRs did not affect NLRP3 inflammasome activation by Fn U112 and nigericin, consistent with reported mouse Nlrp3 results (55). In contrast, the LRR sequence between 717 and 797 was required for Lm activation of the inflammasome, while that between 797 and 1,036 was required for full stimulation by H_2_O_2_. TXNIP, NEK7, and SGT1 interact with elements within the NLRP3 LRRs (717–1,036) (64, 66, 100, 101). Our data demonstrate that elements with the LRRs are differentially involved in responses to known NLRP3 agonists. As the required elements differ, either distinct sets of LRR-associated proteins are required for different stimuli or the function of a common set of proteins varies depending upon the stimuli. That distinct elements within the LRRs are important for some stimuli contrasts with recent work suggesting the LRRs are completely dispensable (55), but two caveats of this study are worth noting. First, mouse Nlrp3 constructs retaining any of the most C-terminal leucine-rich repeats (residues 825–1,033) were not responsive to stimulation with nigericin, alum, and silicon dioxide (55), suggesting a potentially overlooked sensing function for these LRRs. Second, a limited set of agonists types were used and did not include infectious stimuli or those inducing ROS. Importantly, our NLRP3 1–717 construct (lacking all the consensus LRRs and similar to mouse Nlrp3 1–720) was fully responsive to nigericin and *F. novicida*, consistent with the mNlrp3 truncation data, but was unresponsive to *L. monocytogenes* and only partially responsive to H_2_O_2_. In limited experiments, removal of the LRRs after 797 greatly diminished NLRP3 inflammasome activation by monosodium urate (MSU), but activation was partially restored in NLRP3 1-717 (Figure S5), suggesting that 797-1036 are also required for full activation by MSU. Thus, that the LRRs are involved in the response to some NLRP3 stimuli, but dispensable for others, is the most straightforward synthesis of the available data. Further work is needed to establish whether the known NLRP3 associated proteins function to help distinguish between distinct stimuli. Moreover, the divergent responses of given LRR truncations to distinct stimuli demonstrates that a divergent downstream pathway activates the NLRP3 inflammasome and not a single convergent pathway as previously proposed.

The response of specific NLRP3 LRR truncations to individual agonists is disparate and almost certainly due to differences between agonists and/or their evoked cellular signals. However, the capacity of NLRP3 to form an inflammasome in response to diverse stimuli is not abrogated by removal of all the consensus LRRs (NLRP3 1–717). In contrast, truncation beyond 686 (up to 541) abrogates mouse Nlrp3 activation by nigericin, alum, and silicon dioxide (55). Similarly, we observed reduced, but not absent, inflammasome activation after truncation of NLRP3 to 532 (NLRP3 1–532) for H_2_O_2_, nigericin, and MSU. However, while Lm infection did not activate NLRP3 1–717, removing residues 532–717 partially restored inflammasome activation, but had no effect on NLRP3 following infection with Fn U112. Thus, 532–717 is important for NLRP3 inflammasome activation, but acts differentially depending upon the agonist.

While residues 532–717 contain several non-consensus LRR-like elements found in many NLRs (79), it also comprises helical domain (HD) 2 of a potential switch motif similar to that of NLRC4 (102). In NLRC4, a winged helix domain sits between two HD domains (HD1-WHD-HD2) and HD2 facilitates movement between the NACHT and LRR domains to mediate an active “open lock” conformation (102). However, other work suggests that HD1 rather than HD2 facilitates this conformational change (99, 103, 104). NLRP3 1–717, which lacks LRRs but contains HD1-WHD-HD2, was fully responsive to Fn U112 and nigericin suggesting that the open-lock conformation might not be required for NLRP3 activation by these agonists. NLRP3 1–532, which lacks HD2, responded to Lm whereas 1–717 did not, but was less responsive than 1–717 to sterile agonists. These observations suggest that HD2 may function in sensing and distinguishing between agonists. In NLRP3 1–432, the WHD domain is removed leaving only HD1. NLRP3 1432 is responsive to all agonists and removal of the WHD domain improved responsiveness to most when compared with 1–532. Thus, the HD1-WHD-HD2 region may be involved in agonist sensing and may potentially differentiate between sterile and bacterial stimuli. Conformations mediated by this region in the absence of the LRRs may also differ such that inflammasome activation is favored or disfavored depending upon the stimuli. Additionally, if HD1-WHD-HD2 functions primarily to position the LRRs away from the NACHT domain to facilitate activation as proposed, LRR removal should have conferred responsiveness to all agonists. As it did not, this model based on NLRC4 is at least partially incorrect. Finally, since NLRP3 1–432 function still requires ATP-binding, some conformational change is likely and could still involve HD1 (104). Additional attention to how HD1-WHD-HD2 functions may further reveal how NLRP3 is activated by different agonists.

Residues 1–686 of mouse Nlrp3 are reported to be minimally necessary for inflammasome activation by nigericin, alum, and silicon dioxide (55). While we did not specifically test NLRP3 1686, NLRP3 1–532 was responsive to nigericin. Further, NLRP3 1–432 was activated by all stimuli tested and retained ATP-dependence, indicating that the minimally responsive region of NLRP3 is contained within the first 432 residues. Moreover, NLRP3 1–134, completely lacking the NACHT domain and LRRs, is sufficient for an inflammasome response upon H_2_O_2_ stimulation or Fn U112 infection. As an ATP-dependent conformation is required for inflammasome assembly by fulllength NLRP3, residues 1–134 in isolation may adopt an agonist-elicited conformation otherwise prevented by the NBD when ADP-bound or empty. When expressed alone, the pyrin domain of NLRP3 interacts with ASC, but does not form an inflammasome with any stimuli tested. Thus, the previously uncharacterized linker sequence between 94 and 134 is essential. Although ASC is recruited to NLRP3 1–93, once the complex is formed it is unclear why caspase-1 is not recruited/activated. Therefore, the NLRP3 linker region 94–134 functions to initiate caspase-1 activation and suggests cooperativity between the linker and the pyrin domain. The relative inability of the NLRP1 or 2 pyrin domains to substitute for that of NLRP3 suggests that residues 94–134 may cooperates cognately with the NLRP3 pyrin domain to facilitate the inflammasome response. This observation also supports the hypothesis that that NLRP3 PYD and linker domains together participate in agonist sensing.

A disulfide bond between cysteines 8 and 108, spanning the pyrin domain and the linker region, was suggested to be important for NLRP3-ASC interaction (87). However, single point mutants of these cysteines in mNlrp3 are responsive to nigericin (55). C8 and 108 single point mutants in hNLRP3 (this study) similarly fail to diminish the inflammasome response to nigericin, H_2_O_2_, or Fn U112. However, C8 and C108 double mutants reveal these residues together may be important for NLRP3 activation by sterile, but not bacterial, agonists. Although C8 and C108 may cooperate in distinguishing agonists, this pairing does not account for the cooperation between the linker and pyrin domains in general. While our results confirm that C8 and C108 are not forming a double bond required for NLRP3 inflammasome activation [as previously suggested (55)], future studies to explore NLRP3 regulation via cysteine modifications would be informative. The unique chemistry of cysteines supports multiple reversible and irreversible oxidative posttranslational thiol modifications which might allow NLRP3 to function as a molecular switch (105). Copper can form metal ion coordination complexes with cysteines (106) and depletion of bioavailable copper attenuates the NLRP3 inflammasome (107). NLRP3 is also regulated by nitrosylation, however, which of its 43 cysteines are involved remains untested (108). We observed that the antioxidant NAC specifically inhibited H_2_O_2_-elicited NLRP3 inflammasome activation (sterile) but not that with Fn U112 infection (bacterial). As Francisella has an antioxidant system that robustly counteracts cellular ROS, this result is consistent with two distinct activation mechanisms, one potentially based on oxidative modification (perhaps via C8/108) and another that is independent of such modifications and may serve to recognize pathogens that subvert cellular ROS. As sterile NLRP3 agonists and some pathogen elicit cellular ROS, that NLRP3 might distinguish between these is intriguing, but further study is required.

NLRP3 is activated by structurally divergent agonists. Given that NLRP3 agonist-specific responsiveness seems unnecessarily complex, it is widely believed that all NLRP3 agonists engage signaling pathways that converge upon a common downstream intermediate to activate the NLRP3 inflammasome. Since most NLRP3 agonists are known to generate ROS which initiates NLRP3 activation by facilitating NLRP3 association with TXNIP (63, 64, 109), ROS is frequently viewed as a common intermediate. However, IL-1β processing by TXNIP^−/−^ BMDMs in response to islet amyloid polypeptide, MSU, ATP, silicon dioxide, or S. aureus is indistinguishable from wild-type controls (93). Moreover, ROS may only affect the priming step of inflammasome activation (61). Thus, role of ROS and TXNIP in NLRP3 activation is controversial. More recently, the mitotic kinase NEK7 is thought to license NLRP3 inflammasome assembly and activation by ATP and nigericin (63, 64, 66). Thus, NEK7 is considered by some to be a common downstream intermediate for the various agonists. In protein expression studies, NEK7 binds to three major surfaces on NLRP3, namely the LRR, HD2 and nucleotide-binding domains (110). Further, oridonin covalently modifies C279, thereby blocking NEK7 binding to the NLRP3 N-terminal region and reducing inflammasome activity to ATP, nigericin, and MSU (61). As NLRP3 1–432 is required for all of the agonists we tested, except for Fn U112 and H_2_O_2_, C279-dependent NEK7 interaction with NLRP3 1–432 likely occurs in our experiments. However, NLRP3 1–134 does not contain any known interaction surface for NEK7 (66), thus stimuli activating 1–134 (Fn U112 and H_2_O_2_) may not require NEK7 for inflammasome activation. NEK7 binding to NLRP3 is ATPdependent, but whether ATP-binding precedes or follows the interaction is unknown (111). We found that disrupting ATP binding in NLRP3 1–432 impairs the inflammasome response to all NLRP3 agonists tested. Thus, it remains possible that NEK7 interactions are important for all the agonists when the NEK7-NBD binding site is intact, but not when this binding site is absent. Of note, a non-peer reviewed study (pre-print) from the Hornung group indicates that NEK7 is not required for NLRP3 inflammasome activation when TAK1 is activated and suggests that NEK7 might be essential for priming NLRP3, but dispensable for activation (112). While we did not examine TAK1 or related kinase activity in our system, our results may be consistent with NEK7independent activation. Nevertheless, further study using these mutants with diverse agonists may help elucidate a more precise mechanism of NEK7 action. Together, our observations suggest that NEK7 may be a convergent signal pathway for many NLRP3 inflammasome stimuli, while dispensable or functioning differently for others. Collectively, our data demonstrate that different structural features of NLRP3 are required to sense and/or respond to distinct agonists, which provides strong evidence against the current paradigm that a convergent signaling/single intermediate pathway is common to all agonists.

**Figure 6:**
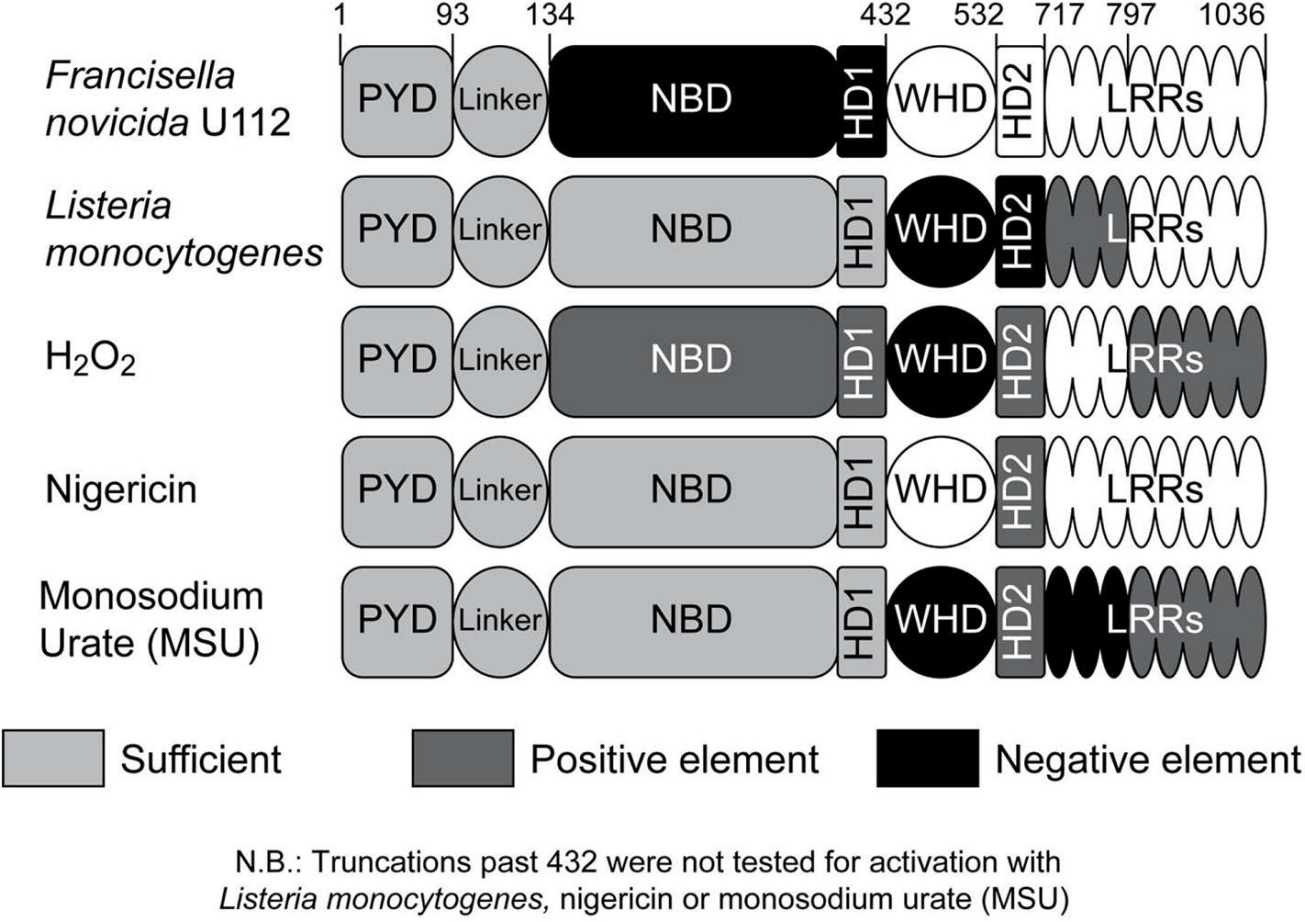
Impact of NLRP3 regions on inflammasome activation by different agonists. Schematic representation of the impact of NLRP3 regions on the inflammasome response to specific agonists; negative effect (black), positive effect (dark gray). The portion of NLRP3 sufficient for inflammasome activation for each agonist is indicated with light gray. Responsive regions for monosodium urate (MSU) are based on limited data (*n* = 2; Figure S5).

In summary, we explored the structural features of NLRP3 required for inflammasome activation by different activation models including K^+^ efflux (nigericin), ROS (H_2_O_2_) and phagosome rupture (Fn U112 and Lm) and found that much of the long-held activation paradigm is likely incorrect. Our data most convincingly challenges the idea that a convergent signaling pathway (e.g., ROS) is shared by all (or most) NLRP3 inflammasome agonists. Instead, these agonists utilize distinct portions of the NLRP3 molecule to effect inflammasome activation and are summarized in Figure 6. Notably, while the LRR domain was initially postulated to act as a ligand sensor (and more recently suggested to be completely dispensable) our data shows that specific regions within the LRR have distinct effects on the response to various ligands. While our data cannot distinguish whether these effects are regulatory in nature or reflect agonist-specific sensing events, some agonists (e.g., Fn U112 and Lm) activate the NLRP3 inflammasome when the canonical NLRP3 LRR domain is absent, while others (e.g. H_2_O_2_ and MSU) exhibit some dependence. Moreover, positing that the LRR functions to maintain NLRP3 in an inactive state *via* intermolecular interaction with the NBD is also incorrect, as even severely truncated mutants maintained an inactive phenotype at rest. The LRR-like region 532–717 of NLRP3 has both negative and positive regulatory functions depending on the agonist further establishing that agonists use functionally distinct mechanisms to activate the NLRP3 inflammasome. Importantly, Fn U112 and H_2_O_2_-mediated NLRP3 inflammasome activation only requires the first 134 amino acids while residues 1–432 are sufficient for activation for all agonists tested. Within these 134 residues cysteine 8 within the pyrin domain and cysteine 108 within the linker domain coordinate activation by sterile agonists (nigericin and H_2_O_2_). Finally, these findings together suggest a new paradigm where the NLRP3 inflammasome is activated via multiple mechanisms, a feature that may allow for future innovative therapeutic interventions.

## Data Availability Statement

All datasets presented in this study are included in the article/Supplementary Material.

## Author Contributions

JH conceptualized and supervised the study. JH and AN designed the experiments, wrote the revised, and edited draft. AN performed homology modeling, generated truncation mutants, analyzed results, and performed inflammasome activation of iBMDMs. TR performed inflammasome reconstitution experiments. KO and AN generated hNLRP3 reconsituted iBMDMs. ED generated truncation and substitution mutants. TR, AN, and JH wrote the original draft and organized the figures. AN and TR performed data and statistical analysis. JH and NS acquired funding for the study. All authors contributed to the article and approved the submitted version.

## Conflict of Interest

The authors declare that the research was conducted in the absence of any commercial or financial relationships that could be construed as a potential conflict of interest.

## Supplementary Material

Figure S1 Structural alignment of NLRP3 and NLRP2 LRRs. **(A)** Table showing the structural template used for LRR homology modeling of NLRPs. LRRs sequence from individual NLRPs were aligned on SWISS MODEL to generate multiple templates for individual LRRs. Templates with over 30% sequence identity were selected for modeling and aligned using PyMOL to calculate root mean square score (rms) compared to NLRP3 LRRs. NLRP2 LRRs had the lowest rms score with more than 80% coverage and was selected to substitute for those of NLRP3. **(B)** Aligned homology models of the LRRs of NLRP3 (black) and NLRP2 (gray). Details of alignment and template characteristics in tabular form (left). Images from rotation of aligned models on the X- and Y- axis with the respective degree of rotation on individual axes (right).

Figure S2 Characterization of NLRP3 N-terminal region. **(A)** Schematic of the exon structure coding linker domains for all NLRPs. **(B)** Multiple sequence alignment of Pyrin domain and linker region for NLRPs known to interact with ASC. **(C)** Multiple sequence alignment of NLRP3 across different species (Red bar indicates the Pyrin domain). **(D)** Fluorescence imaging and quantification of specks in HEK293T cells expressing myc-ASC and NLRP3 or NLRP3 1–93.

Figure S3 Structural alignment of NLRP1, NLRP2, and NLRP3 PYD. **(A)** Multiple sequence alignment of the Pyrin domains of NLRP1, 2, and 3 with sequence identities and similarities. **(B,C)** Aligned homology models of the **(B)** Pyrin domains of NLRP3 (red) and NLRP1 (blue) and **(C)** the NLRP3 (red) and NLRP2 (green) LRRs. Details of alignment and template characteristics in tabular form (top). Images from rotation of aligned models on the X- and Y- axis with respective degree of rotation on individual axes (bottom).

Figure S4 Human NLPR3 does not substitute for mouse NLRP3. **(A)** IL-1β production by WT, mNlrp3-/-, or human NLRP3 expressing mNlrp3-/- immortalized mouse macrophages after stimulation with LPS (100 ng/ml; 4 h) and 5 μM nigericin (2 h). Inset: Western blot for NLRP3 in the indicated cells. Data represent the mean ± *SD* for two independent experiments. **(B)** IL-1β production by HEK293T cells expressing human ASC, Caspase-1, and IL-1β and the indicated human or mouse NLRPs were infected with Fn U112 (MOI = 50). Mean ± *SD* are shown from one preliminary experiment. **(C)** IL-1β production by WT, mNlrp3-/-, or human NLRP3 expressing mNlrp3-/- immortalized mouse macrophages after stimulation with LPS (100 ng/ml; 11 h) and 5 μM nigericin (1 h). Data represent the mean ± *SD* for three independent experiments. **(D)** Percent cell death for cells in **(C)**. Mean ± *SD* for three independent experiments.

Figure S5 Monosodium urate (MSU) display distinct activation requirements. **(A)** Schematic representation of C-terminal LRR domain and NBD domain truncation mutants. **(B)** IL-1β response of HEK293T cells expressing ASC, pro-Caspase-1, pro-IL-1β, and NLRP3 or the indicated mutants after stimulation with MSU (150 μg/ml) for 1 h. Supernatant IL-1β was measured by ELISA. Means +/− SEM are shown (*n* = 2).
