# Supplementary figures and images for: NLRP3 Sensing of Diverse Inflammatory Stimuli Requires Distinct Structural Features
Source: Front Immunol. 2020 Aug 26;11:1828. doi: 10.3389/fimmu.2020.01828 (PMC7479093; doi:10.3389/fimmu.2020.01828)

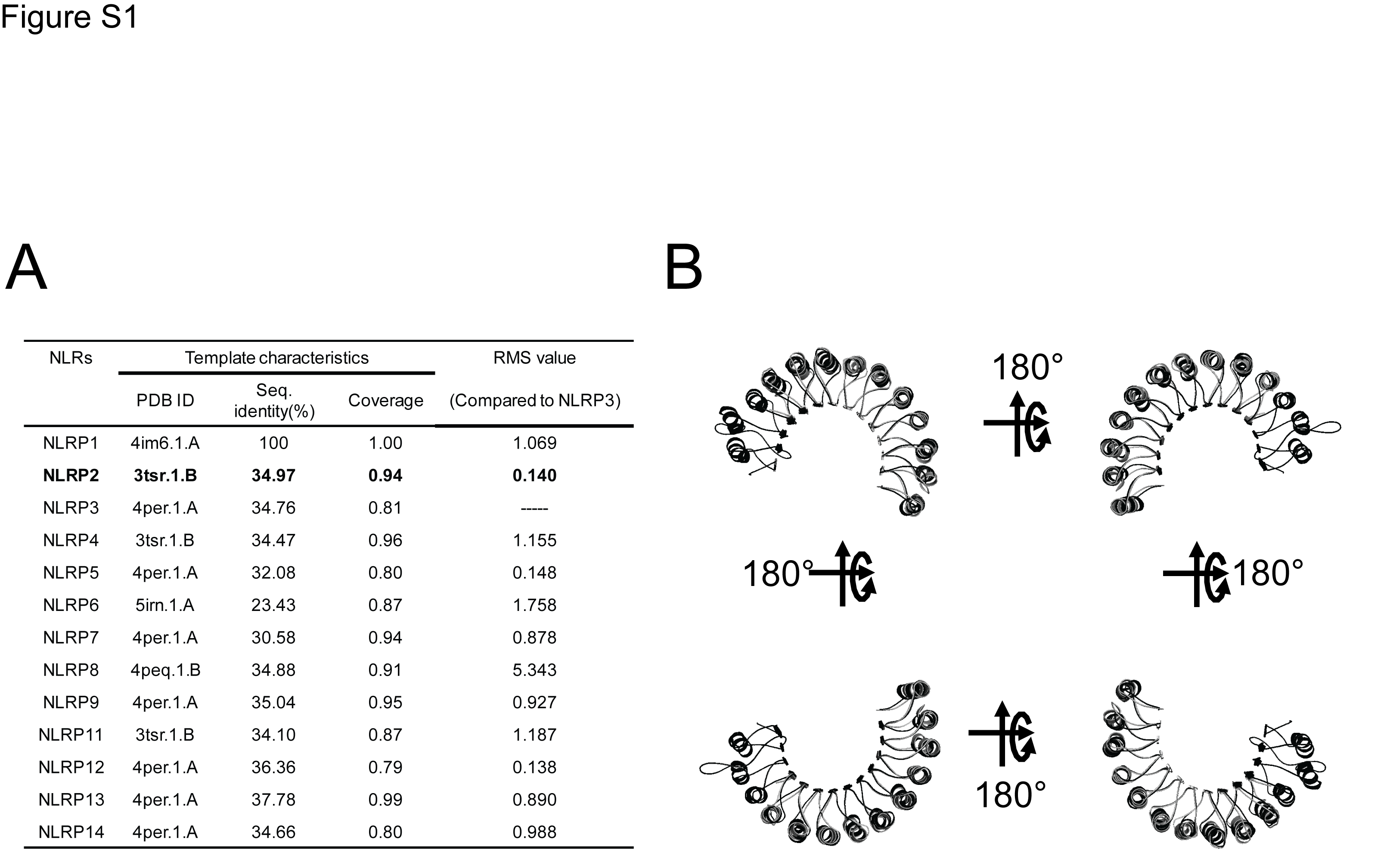

Supplement: Figure S1 — Structural alignment of NLRP3 and NLRP2 LRRs. (A) Table showing the structural template used for LRR homology modeling of NLRPs. LRRs sequence from individual NLRPs were aligned on SWISS MODEL to generate multiple templates for individual LRRs. Templates with over 30% sequence identity were selected for modeling and aligned using PyMOL to calculate root mean square score (rms) compared to NLRP3 LRRs. NLRP2 LRRs had the lowest rms score with more than 80% coverage and was selected to substitute for those of NLRP3. (B) Aligned homology models of the LRRs of NLRP3 (black) and NLRP2 (gray). Details of alignment and template characteristics in tabular form (left). Images from rotation of aligned models on the X- and Y- axis with the respective degree of rotation on individual axes (right). [file Image_1.TIF]

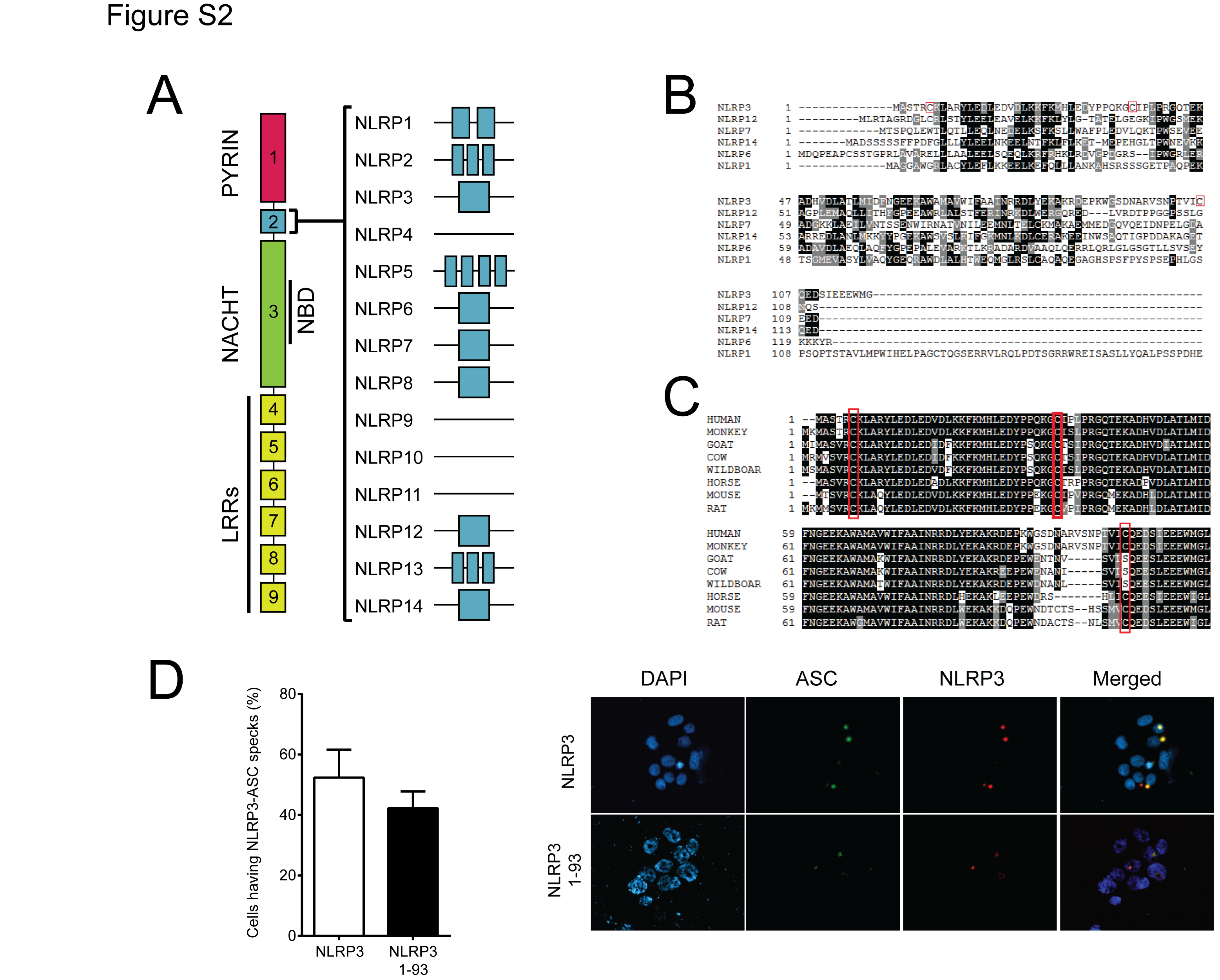

Supplement: Figure S2 — Characterization of NLRP3 N-terminal region. (A) Schematic of the exon structure coding linker domains for all NLRPs. (B) Multiple sequence alignment of Pyrin domain and linker region for NLRPs known to interact with ASC. (C) Multiple sequence alignment of NLRP3 across different species (Red bar indicates the Pyrin domain). (D) Fluorescence imaging and quantification of specks in HEK293T cells expressing myc-ASC and NLRP3 or NLRP3 1–93. [file Image_2.TIF]

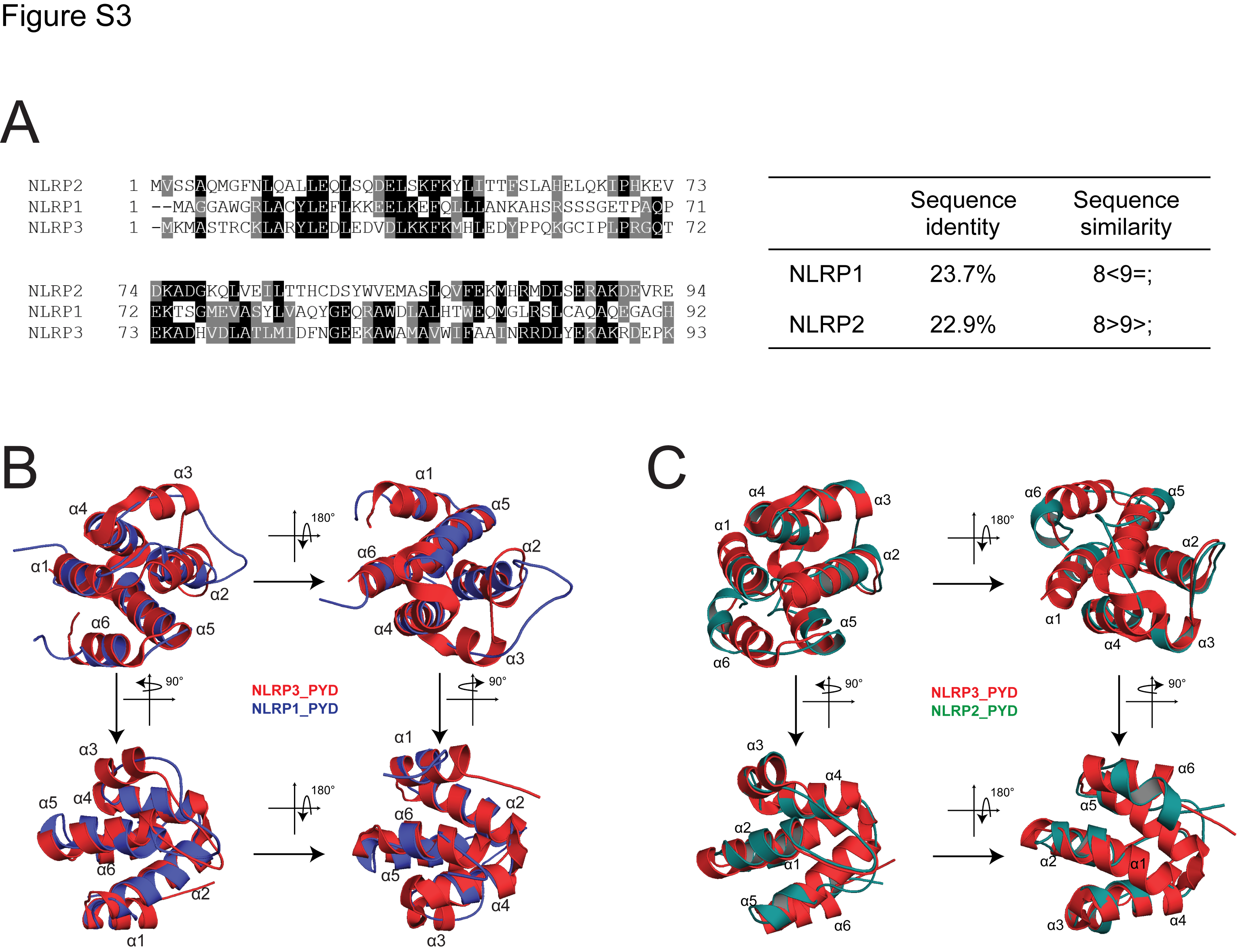

Supplement: Figure S3 — Structural alignment of NLRP1, NLRP2, and NLRP3 PYD. (A) Multiple sequence alignment of the Pyrin domains of NLRP1, 2, and 3 with sequence identities and similarities. (B,C) Aligned homology models of the (B) Pyrin domains of NLRP3 (red) and NLRP1 (blue) and (C) the NLRP3 (red) and NLRP2 (green) LRRs. Details of alignment and template characteristics in tabular form (top). Images from rotation of aligned models on the X- and Y- axis with respective degree of rotation on individual axes (bottom). [file Image_3.TIF]

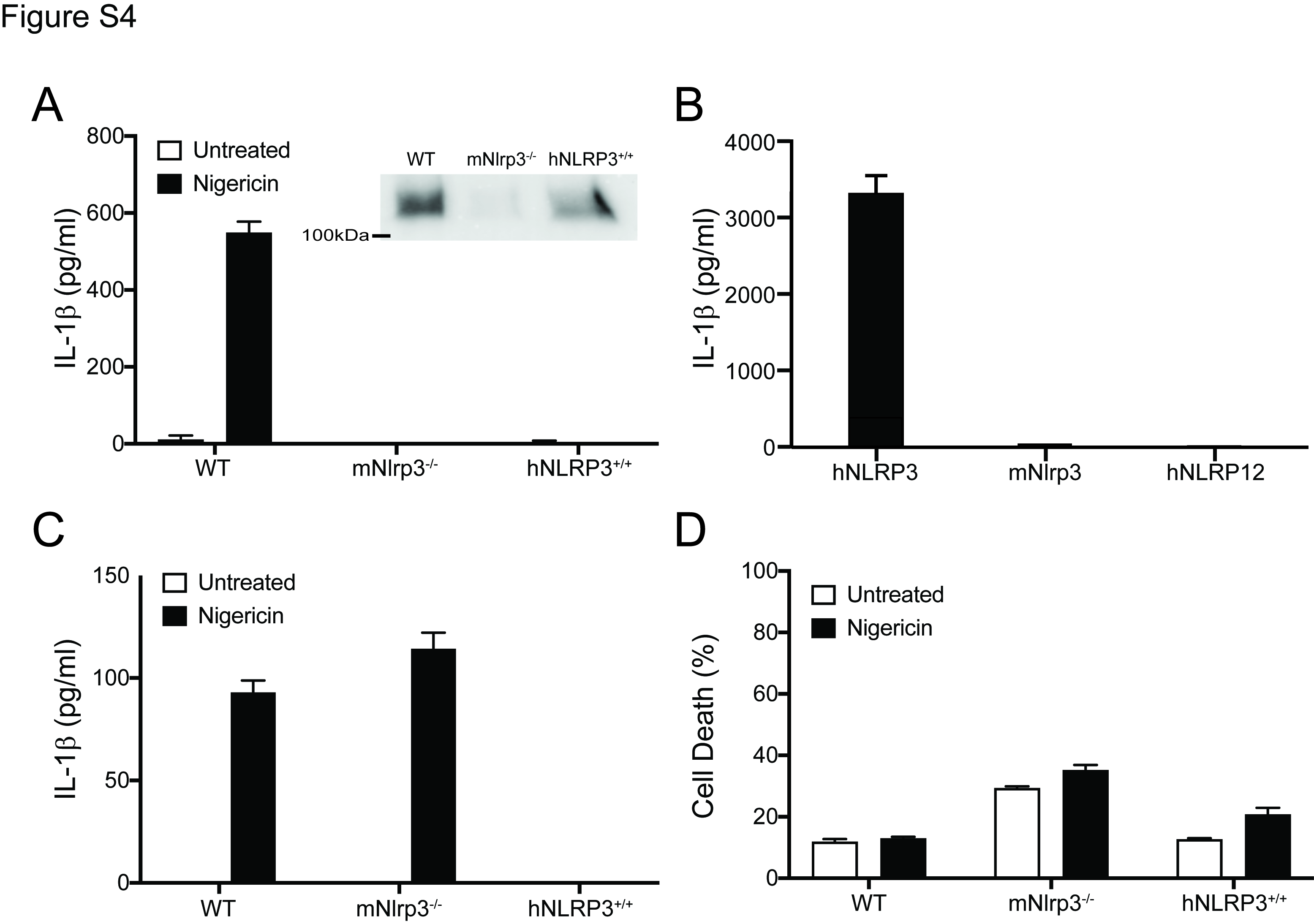

Supplement: Figure S4 — Human NLPR3 does not substitute for mouse NLRP3. (A) IL-1β production by WT, mNlrp3-/-, or human NLRP3 expressing mNlrp3-/- immortalized mouse macrophages after stimulation with LPS (100 ng/ml; 4 h) and 5 μM nigericin (2 h). Inset: Western blot for NLRP3 in the indicated cells. Data represent the mean ± SD for two independent experiments. (B) IL-1β production by HEK293T cells expressing human ASC, Caspase-1, and IL-1β and the indicated human or mouse NLRPs were infected with Fn U112 (MOI = 50). Mean ± SD are shown from one preliminary experiment. (C) IL-1β production by WT, mNlrp3-/-, or human NLRP3 expressing mNlrp3-/- immortalized mouse macrophages after stimulation with LPS (100 ng/ml; 11 h) and 5 μM nigericin (1 h). Data represent the mean ± SD for three independent experiments. (D) Percent cell death for cells in (C). Mean ± SD for three independent experiments. [file Image_4.tif]

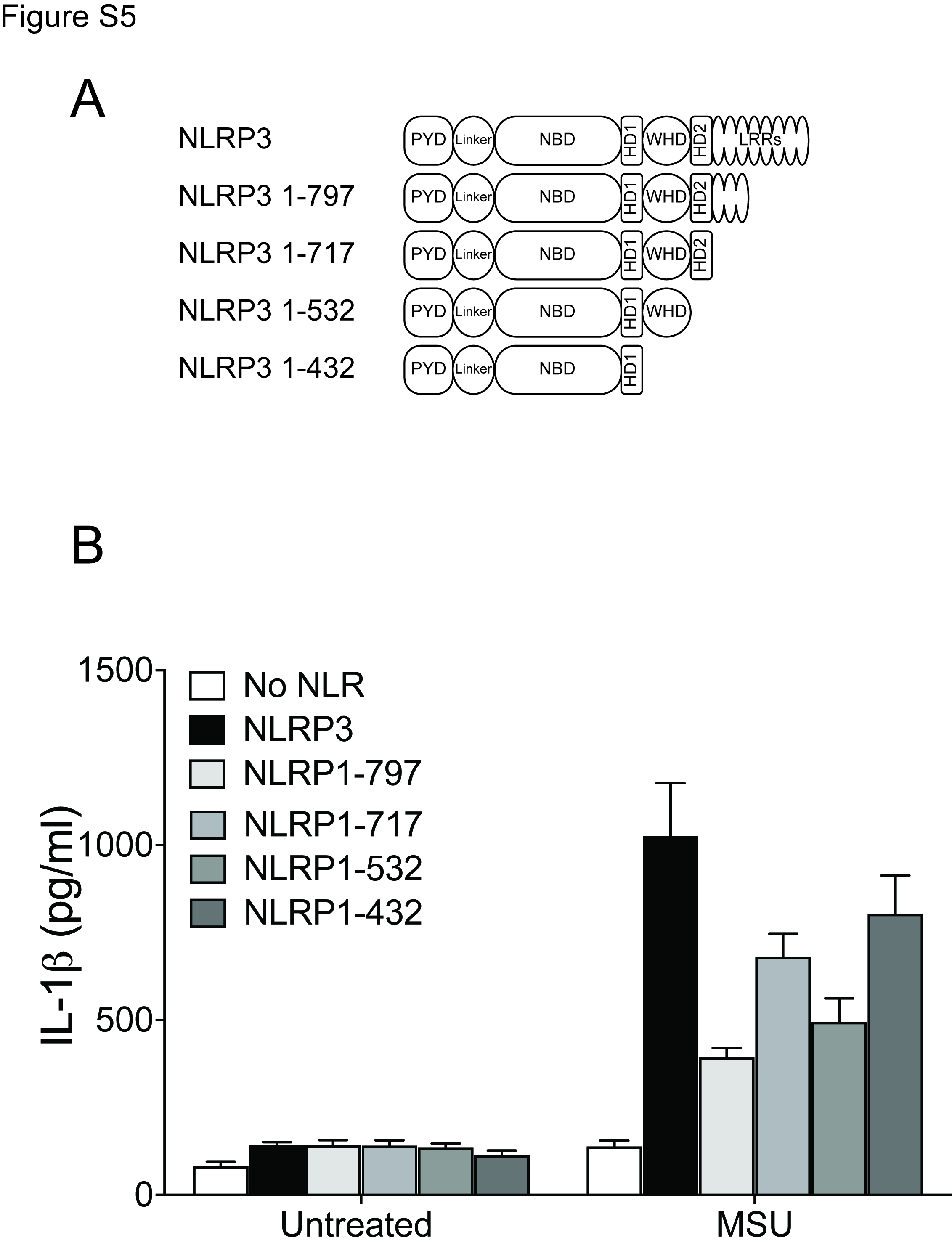

Supplement: Figure S5 — Monosodium urate (MSU) display distinct activation requirements. (A) Schematic representation of C-terminal LRR domain and NBD domain truncation mutants. (B) IL-1β response of HEK293T cells expressing ASC, pro-Caspase-1, pro-IL-1β, and NLRP3 or the indicated mutants after stimulation with MSU (150 μg/ml) for 1 h. Supernatant IL-1β was measured by ELISA. Means +/− SEM are shown (n = 2). [file Image_5.tif]
